# Importance of conserved hydrophobic pocket region in yeast mitoribosomal mL44 protein for mitotranslation and transcript preference

**DOI:** 10.1016/j.jbc.2024.107519

**Published:** 2024-06-29

**Authors:** Jodie M. Box, Margo E. Higgins, Rosemary A. Stuart

**Affiliations:** Department of Biological Sciences, Marquette University, Milwaukee, Wisconsin, USA

**Keywords:** mitochondria, ribosome, mitoribosome, MRPL44, MrpL3/mL44, oxidative phosphorylation, yeast, protein synthesis

## Abstract

The mitochondrial ribosome (mitoribosome) is responsible for the synthesis of key oxidative phosphorylation subunits encoded by the mitochondrial genome. Defects in mitoribosomal function therefore can have serious consequences for the bioenergetic capacity of the cell. Mutation of the conserved mitoribosomal mL44 protein has been directly linked to childhood cardiomyopathy and progressive neurophysiology issues. To further explore the functional significance of the mL44 protein in supporting mitochondrial protein synthesis, we have performed a mutagenesis study of the yeast mL44 homolog, the MrpL3/mL44 protein. We specifically investigated the conserved hydrophobic pocket region of the MrpL3/mL44 protein, where the known disease-related residue in the human mL44 protein (L156R) is located. While our findings identify a number of residues in this region critical for MrpL3/mL44’s ability to support the assembly of translationally active mitoribosomes, the introduction of the disease-related mutation into the equivalent position in the yeast protein (residue A186) was found to not have a major impact on function. The human and yeast mL44 proteins share many similarities in sequence and structure; however results presented here indicate that these two proteins have diverged somewhat in evolution. Finally, we observed that mutation of the MrpL3/mL44 does not impact the translation of all mitochondrial encoded proteins equally, suggesting the mitochondrial translation system may exhibit a transcript hierarchy and prioritization.

ATP, the vital energy source for the cell's diverse metabolic needs, is produced by mitochondria through the process of oxidative phosphorylation (OXPHOS) (1, 2, 3, 4, 5). The survival and vitality of many organisms is therefore dependent on the mitochondria's capacity to aerobically synthesize ATP. Embedded within the mitochondrial inner membrane, OXPHOS enzymes are oligomeric complexes composed of proteins of both nuclear and mitochondrial genetic origin. The majority of OXPHOS subunits are encoded on nuclear genes, synthesized on cytosolic ribosomes, and imported into the organelle, where they functionally assemble with their mitochondrial-encoded partners into OXPHOS complexes. A small, but important, number of the OXPHOS core enzymatic subunits are encoded on the mitochondrial genome (mtDNA) and synthesized by mitochondrial ribosomes (mitoribosomes) located in the mitochondrial matrix and tethered to the inner membrane. In the yeast *Saccharomyces cerevisiae* (*S. cerevisiae*), the model organism of this study, the mtDNA encodes components of the cytochrome *bc*_1_, cytochrome *c* oxidase (COX), and F_1_F_o_-ATP synthase enzymes (6). The biogenesis of these OXPHOS enzymes is therefore dependent on mitochondria having functioning mitoribosomes. Consequently, defects in the mitotranslational process have the potential to negatively impact OXPHOS complex assembly and thus the bioenergetic capacity of the cell (7, 8, 9, 10, 11, 12, 13, 14, 15, 16). Eukaryotic cells dependent on aerobic respiration for their differentiation and/or viability are therefore gravely affected by a dysfunction in their mitoribosomal systems. The molecular analysis of mitoribosomes, their assembly, function, and regulation is therefore crucial to our understanding of eukaryotic cell viability and metabolic regulation.

The mitoribosomes are compositionally distinct from their cytosolic counterparts and are composed of rRNA encoded by the mtDNA and proteins which, except for Var1 of the small mitoribosomal subunit in the *S. cerevisiae*, are encoded by nuclear genes. Mammalian mitoribosomes also contain a structural tRNA component (17, 18, 19, 20). Mitochondrial protein translation disorders have been shown to be directly associated with a wide range of pathophysiological conditions and can include early-onset cardiomyopathy, neuromuscular, and progressive multisystem deficiencies (13, 14, 15, 21, 22, 23, 24, 25). Investigating the composition, structure, function, and regulation of mitoribosomes can therefore have major importance for our molecular understanding of the pathophysiology of these mitochondrially based disease states. Some mitoribosomal translation defects have their genetic origin in the mtDNA and for example can include mutations in genes encoding OXPHOS protein components, tRNAs, and/or mitoribosomal rRNAs. On the other hand, mutations in nuclear-encoded genes can also genetically underlie mitochondrial translation defects, for example, in genes encoding mitochondrial aminoacyl-tRNA synthetases and tRNA-modifying enzymes, the mitoribosomal protein components themselves, and/or nuclear-encoded protein factors involved in mitotranslation initiation, elongation, and termination.

The nuclear-encoded mitoribosomal protein mL44 is a component of the large mitoribosomal subunit conserved from fungi through humans. The mL44 protein is termed a mitospecific mitoribosomal protein because the mL44 protein family is found only in mitochondrial ribosomes and not present in their prokaryotic ancestors, the bacterial ribosome (17, 18, 26, 27). The mitospecific nature of the mL44 protein family suggests that this protein may play an important structural and/or regulatory role unique to mitochondrial ribosomes, rather than playing an integral part in the protein synthesis peptidyltransferase reaction *per se*. The mL44 protein is located in the membrane protuberance region of mitoribosomes adjacent to the polypeptide exit site where the nascent chain emerges from the ribosome (Fig. S1*A*) (17, 18, 20, 26, 27, 28, 29). The membrane protuberance region has been proposed to support the membrane anchoring of mitoribosomes, which enables the cotranslational insertion of the resulting nascent chains into the mitochondrial inner membrane (20). Mitochondrial ribosomes are assembled directly and are permanently tethered to the membrane and unlike bacterial ribosomes, do not cycle on and off the membrane (12, 30, 31). Mutation of the human MRPL44/mL44 gene has been reported to be directly linked to childhood-onset hypertrophic cardiomyopathy and progressive multi-system disease (13, 14, 15, 32, 33). The MRPL44/mL44 mutation underlying this pathophysiological presentation was shown to be a homozygous missense mutation in codon 156 encoding the hydrophobic residue Leu(L)156 and where it was mutated to encode the polar Arg(R) residue and termed the L156R mutation (13, 14). Measurements of OXPHOS levels in fibroblasts isolated from patients bearing the homozygous L156R mutation indicated they displayed strongly decreased OXPHOS capacity, in particular levels of the terminal COX enzyme (complex IV, COX) (13, 15, 32). A reduction in mitochondrial translation output levels was also reported for cells harboring the MRPL44/mL44 L156R mutation (15). Although these clinical studies highlight the importance of MRPL44/mL44 protein, the molecular basis for the defect underlying the measured loss of OXPHOS activity caused by the L156R mutation, remains unknown.

In this study, we have analyzed the yeast *S. cerevisiae* mL44 protein, which is termed MrpL3/mL44. The MrpL3/mL44 protein has been previously demonstrated to be required for mitotranslation and thus for cellular respiration in yeast (31). The yeast and human mL44 proteins share sequence similarity, and available cryoEM analysis of both yeast and mammalian mitoribosomes indicate that they also share considerable structural similarity. The pathogenic mL44 mutation L156R is located in a hydrophobic pocket region conserved between all mL44 proteins (17, 18, 20, 27). The yeast system provides genetic and biochemical advantages to probe the molecular consequences of disease-related mitoribosomal mutations. Due to their ability to thrive under fermentation conditions when grown on suitable carbon sources (*e.g.*, glucose or galactose), respiratory-deficient yeast mutants caused by impaired mitoribosomal function remain viable when grown under fermentation conditions, enabling their further biochemical analysis. In this study, we have created a genetic system to analyze the yeast MrpL3/mL44 protein and to model the L156R mutation of the human MRPL44/mL44 protein in its yeast counterpart. We report here that while yeast MrpL3/mL44 and human MRPL44/mL44 proteins share many similarities, the corresponding disease-related mutation, L156R, does not greatly impact the function of the yeast MrpL3/mL44 protein. Furthermore, we have used the yeast MrpL3/mL44 genetic system to further probe conserved aspects of the hydrophobic pocket of the mL44 proteins where the disease-related mutation is located. We report here on the isolation of a number of temperature-sensitive mutations in the MrpL3/mL44 protein, which significantly impair the respiratory viability of the cell at stress temperatures. Our results demonstrate the importance of the conserved hydrophobic pocket region of the mL44 protein for supporting the assembly and hence translational activity of the mitoribosome. Our analysis also uncovered mutations of the MrpL3/mL44 that result in a novel phenotype, elevated levels of Cox2 synthesis, and COX complex accumulation, which was correlated with decreased cellular viability under fermentation conditions. Finally, our findings shed important light on how the human and yeast mitoribosomes may have evolutionarily diverged with respect to their mL44 proteins and membrane protuberances.

## Results

### The hydrophobic nature of residue Ala(A)186 of MrpL3/mL44, equivalent to Leu(L)156 of human MRPL44/mL44, is not required for function

**Figure 1:**
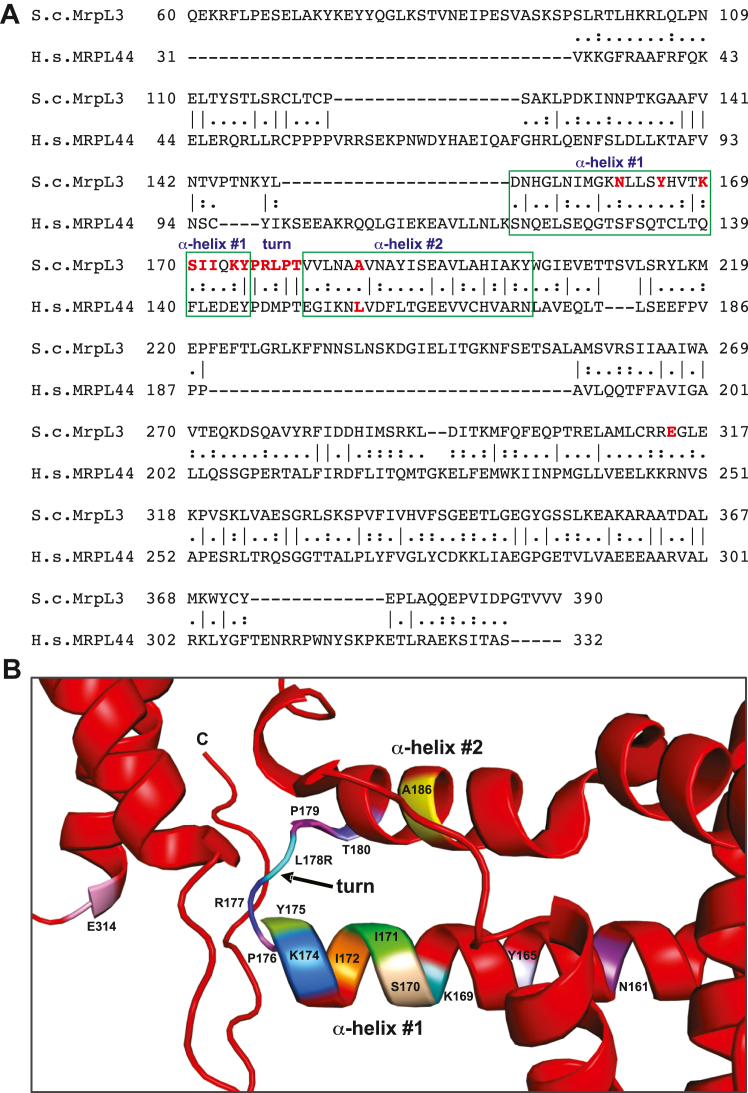
**Identification and mutation of conserved residues in MrpL3/mL44.***A*, sequence alignment of select mL44 family members was performed with CLUSTAL O, with amino acid residue numbers indicated. Sequences used are as follows: S.c. MrpL3, *S. cerevisiae* MrpL3/mL44 (UniProtKB: P36516) and H.s. MRPL44, *Homo sapiens* (human) MRPL44/mL44 (UniProtKB: Q9H9J2). Residues corresponding to the α-helix #1-turn-α-helix #2 region are indicated. Conservation between groups of residues with strongly similar properties are indicated by the CLUSTAL symbol “:”, whereas identical residues are indicated by the CLUSTAL “|” symbol. The CLUSTAL “.” symbol indicates conservation between residues of weakly similar properties. Residues in *red* type in the *S.c.* MrpL3/mL44 sequence indicate those targeted for mutagenesis. *B*, PyMOL image of *S. cerevisiae* MrpL3/mL44 protein “α-helix #1-turn-α-helix #2” region (from PDB 5MRC) with the amino acid residues targeted for mutagenesis indicated in color and labeled with one-letter residue code and number.

In the human MRPL44/mL44, the disease-related residue L156 is located in a highly hydrophobic pocket environment, largely composed of two adjacent alpha (α)-helical segments, with hydrophobic aspects of the mL44 protein (13, 20). The α-helical segment arrangement (termed here “α-helix #1-turn-α-helix #2”) with its formed hydrophobic pocket are elements conserved between the mammalian mL44 proteins and their yeast homolog MrpL3/mL44 (Fig. S1*B*). The introduction of a charged residue through the disease related L156R mutation has been viewed as incompatible with the hydrophobic nature of this mL44 pocket and predicted to cause a perturbation in the overall structure of the MRPL44/mL44 protein (13, 20). In the yeast MrpL3/mL44 protein, the α-helix #1-turn-α-helix #2 region is comprised of residues 151-202 (Fig. 1, *A* and *B*). Sequence alignment of yeast MrpL3/mL44 and human MRPL44/mL44 indicates that residue Ala(A)186 of MrpL3/mL44, a small hydrophobic residue, is present in the equivalent position of L156 from the human MRPL44/mL44 protein (Fig. 1, *A* and *B*).

**Figure 2:**
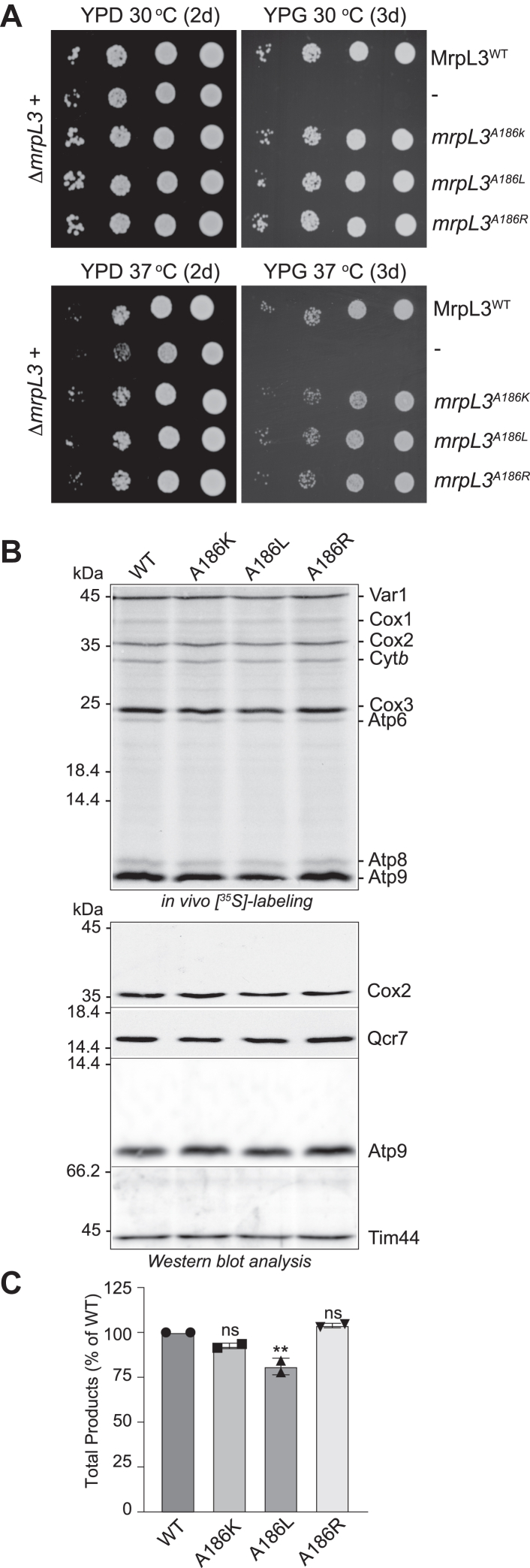
**The hydrophobic nature of residue Ala(A)186 of yeast MrpL3/mL44, equivalent to Leu(L)156 of human MRPL44/mL44, is not required for function in yeast.***A*, 10-fold serial dilutions (dilutions from *right* to *left*) of Δ*mrpL3* strains with pRS413 plasmid containing no insert (−) or a gene insert encoding the WT MrpL3/mL44 protein (MrpL3^WT^) or a mutated *mrpL3* derivative, as indicated. Strains were taken from 24 h growths on selective SD plates and spotted onto YP plates containing either glucose (YPD) (2 d) or glycerol (YPG) (3 d) and incubated at either 30 °C or 37 °C. *B*, the indicated *mrpL3* strains adapted to glycerol-selective synthetic media and were grown to mid-log phase at 37 °C, as described in Experimental procedures. Mitochondrial translation capacity was analyzed *in vivo* for 10 min in the presence of cycloheximide and [^35^S]methionine. Cells were isolated, solubilized, and proteins were analyzed by SDS-PAGE and Western blotting, followed by autoradiography (*upper* panels). The equivalent of A_600_ 0.3 of cells were loaded on each gel. Steady state levels of OXPHOS proteins Cox2, Qcr7, and Atp9, and Tim44 (loading control) from the same cell extracts were also analyzed by Western blotting and immunedecoration with indicated antibodies (*lower* panels). The steady state OXPHOS subunit levels data from independent Western blots were also quantified and summarized in Fig. S2. *C*, the total amount of proteins synthesized during the *in vivo* radiolabeling period for each strain (*i.e.*, sum of Var1, Cox1, Cox2, Cox3, Cyt*b*, Atp6, Atp8, and Atp9 synthesis signals) was quantified by phosphorimaging or ImageJ analysis of resulting autoradiographs and expressed as a percentage of the WT MrpL3/mL44 control. Data from technical replicates (n = 2) are provided. Bars indicate the means ± SD. The significance between measurements was determined by one-way ANOVA with Dunnett’s *post hoc* test using GraphPad Prism 9. Comparison of each *mrpL3* mutant to its respective WT control resulted in either no significant difference (ns) or in a significant difference, as indicated by ∗∗*p* < 0.01. WT, wild-type MrpL3/mL44; A186K, *mrpL3*^A186K^; A186L, *mrpL3*^A186L^; A186R, *mrpL3*^A186R^.

The functional significance of the α-helix #1-turn-α-helix #2 region of mL44 was addressed in this study using the yeast MrpL3/mL44 protein as a model system. The disease-equivalent residue located in this region, that is, A186 of yeast MrpL3/mL44, was initially targeted for site-directed mutagenesis. We created three A186 mutant *mrpL3* derivatives, where the codon encoding A186 was mutated either to one encoding a positively charged residue Arg(R) or Lys(K) residue (to mimic the L156R disease mutation of MRPL44/mL44) or exchanged with one encoding another hydrophobic residue, Leu(L) (to resemble the WT human MRPL44/mL44 residue L156, thereby retaining the hydrophobic nature of the pocket). The A186 mutant *mrpL3* derivatives were expressed in yeast and tested for their ability to functionally substitute for the WT MrpL3/mL44 protein (Fig. 2). The resulting strains, *mrpL3*^A186R^, *mrpL3*^A186K^, and *mrpL3*^A186L^ were first grown on the nonfermentable carbon source glycerol to test for their ability to support oxidative phosphorylation-based cellular metabolism. Consistent with previous reports (31), the presence of MrpL3/mL44 was found to be required for yeast respiratory-based growth (Fig. 2*A*). A serial dilution-based growth analysis indicated that all three *mrpL3* mutant strains appeared fully capable of supporting respiratory-based yeast growth at the optimal and stress growth temperatures for yeast, 30 °C and 37 °C, respectively (Fig. 2*A*). Fermentation-based growth (glucose media) also appeared normal for the *mrpL3* mutant strains and closely mirrored that of the corresponding MrpL3/mL44 WT control analyzed in parallel.

Mitochondrial translation capacities of the *mrpL3* A186 strains were next directly analyzed through *in vivo* radiolabeling in the presence of [^35^S]methionine (and additional cycloheximide to inhibit cytosolic ribosomal activity) (Fig. 2*B*). Analysis was performed on cells grown in glycerol/ethanol media (OXPHOS-demanding conditions) and at 37 °C, an elevated temperature to stress the cells with the hope of teasing out a translational phenotype if it existed in the *mrpL3* A186 mutants. The synthesis of each of the mtDNA-encoded proteins, Var1 (a component of the small ribosomal subunit), Cox1, Cox2, and Cox3 of the COX complex, cytochrome *b* of the cytochrome *bc*_1_ enzyme, and F_o_-ATP synthase subunits Atp6, Atp8, and Atp9, was observed in the three *mrpL3* A186 mutants analyzed. Both the levels and profiles of radiolabeled proteins synthesized in the *mrpL3*^A186R^, *mrpL3*^A186K^, and *mrpL3*^A186L^ strains were found to be similar to those of the control WT MrpL3/mL44 cells, suggesting that mitotranslation was not limiting for OXPHOS assembly in these mutants (Fig. 2*B*, upper panel and Fig. 2*C*). Western blotting and immunedecoration of the resulting cell extracts with antibodies against key marker subunits of the OXPHOS enzymes confirmed this. The steady state levels of Cox2 (COX complex), Qcr7 (cytochrome *bc*_1_ complex), and the F_o_-subunit Atp9 (F_1_F_o_-ATP synthase) indicated that the cellular levels of the OXPHOS enzymes in all three of the *mrpL3* A186 mutants appeared similar to those of the MrpL3/mL44 WT control (Fig. 2*B*, lower panel and Fig. S2). The cellular level of these specific OXPHOS subunits was used as an indicator of OXPHOS complex levels in this study, as each of the marker proteins analyzed are proteolytically turned over if the assembly of their respective OXPHOS complex is defective (34, 35, 36).

Although the equivalent mutation (L156R) in the human protein has been reported to be detrimental for the function of the human MRPL44/mL44 protein, we conclude here that the substitution of the hydrophobic residue A186 of MrpL3/mL44 for a positively charged one, Arg(R) or Lys(K) within this conserved hydrophobic pocket region, did not inhibit the ability of the yeast MrpL3/mL44 protein to support mitochondrial translation or OXPHOS activity under respiratory growth conditions.

### The human MRPL44/mL44 protein cannot functionally replace the yeast MrpL3/mL44 protein

**Figure 3:**
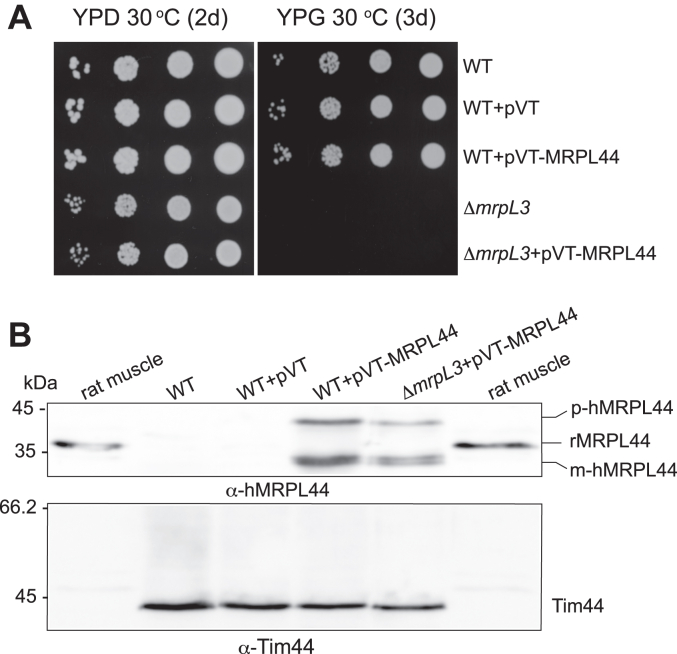
**The human MRPL44/mL44 protein cannot functionally replace the yeast MrpL3/mL44 protein to support aerobic respiration in yeast.***A*, growth phenotype analysis on glucose (YPD) (2d) or glycerol (YPG) (3d) was performed, as described in Fig. 2*A*. Strains used included the W303-1A WT yeast cells harboring either the empty pVT100U-ADH1 plasmid (WT+pVT) or the same plasmid containing the cloned the human MRPL44 gene (WT+pVT-MRPL44). The Δ*mrpL3* mutant and the Δ*mrpL3* mutant harboring the pVT100U-ADH1-MRPL44 plasmid (Δ*mrpL3*+pVT-MRPL44) were analyzed in parallel. *B*, mitochondria isolated from the above strains grown on glucose media were analyzed by SDS-PAGE analysis and Western blotting. Immunedecoration using a human MRPL44 monoclonal antibody that broadly recognizes mammalian MRPL44 species was performed. A rat muscle cell extract was also analyzed on the SDS-PAGE in parallel to control for the functionality of the mammalian MRPL44 antibody. Tim44 levels were also analyzed (as a loading control for the yeast mitochondria). The p-hMRPL44 and m-hMRPL44 labels indicate the putative precursor and mature species of the human MRPL44 protein, while rMRPL44 indicates the rat MRPL44 protein.

The finding that the mutation of the A186 residue of the yeast MrpL3/mL44 protein did not compromise yeast mitoribosomal function suggested there may be evolutionary differences between the human and yeast mL44 proteins. We next tested if the human MRPL44/mL44 protein could functionally replace the yeast MrpL3/mL44 protein in its ability to support yeast aerobic respiration. To do so, we cloned the ORF encoding the human MRPL44/mL44 protein into a multi-copy (pVT100U-ADH1) yeast expression plasmid (37), which had been engineered to contain the strong ADH1 constitutive promoter. Expression of the human MRPL44/mL44 protein in the WT yeast cells did not appear to functionally interfere with the endogenous MrpL3/mL44 protein, as the presence of the human MRPL44/mL44 protein was observed not to be toxic to the cell under nonfermentative growth conditions (Fig. 3*A*). Successful expression of the human MRPL44/mL44 protein in the transformed WT cells was confirmed following Western blotting of cells extracts with a MRPL44 monoclonal antibody that broadly recognizes mammalian MRPL44 species (Fig. 3*B*). Two differentially migrating forms of human MRPL44/mL44 protein were observed following SDS-PAGE/Western blotting in the yeast cells, of which the smaller form was consistent in size of the mature MRPL44/mL44 species, that is, the form generated following removal of its cleavable N-terminal mitochondrial targeting sequence. The larger species observed may represent the unprocessed MRPL44 precursor species, indicating that the human mitochondrial targeting sequence of MRPL44/mL44 may not be efficiently cleaved by the yeast mitochondrial processing peptidase following import into mitochondria. The ability of the human MRPL44/mL44 protein to support the respiratory-based growth in the absence of the yeast MrpL3/mL44 protein was next tested following the genetic disruption of the endogenous yeast MRPL3 gene in the cells expressing the human MRPL44/mL44 protein (Fig. 3*A*). The resulting Δ*mrpL3* strain expressing human MRPL44/mL44 protein failed to grow on glycerol containing media, indicating the inability of the human MRPL44/mL44 protein to complement the Δ*mrpL3* respiratory-deficient phenotype (Fig. 3, *A* and *B*). A similar result was also obtained, when the human MRPL44/mL44 protein was independently expressed using a different yeast expression system, an integrating Yip352 plasmid engineered to contain the strong galactose-inducible GAL10 promoter (results not shown). We conclude therefore that although the yeast and MrpL3/mL44 and human MRPL44/mL44 proteins share sequence and structural similarities, they appear to have diverged somewhat in their evolution such that the human mL44 protein cannot functionally substitute for its yeast counterpart.

### Residues R177 and L178 of the α-helix#1-turn-α-helix#2 region of MrpL3/mL44 are critical for function

To further probe the hydrophobic pocket region in which residue A186 of MrpL3/mL44 is located, we individually mutated other residues within the α-helix#1-turn-α-helix#2 region of MrpL3/mL44. Our mutagenesis approach initially focused largely on residues immediately prior to and within the “turn” aspect linking both α-helical segments (encompassed by residues 169 through 180 of MrpL3/mL44) (Fig. 1, *A* and *B*). Targeted first were the two proline (Pro,P) residues (P176 and P179) considered to be structurally important for creating the bends of the turn region linking α-helices #1 and #2 and represent residues highly conserved between diverse mL44 proteins. Residues P17Six and P179 (equivalent to P146 and P149 of human MRPL44/mL44) were individually mutated to alanine residues. Growth analysis of the resulting mutants, the *mrpL3*^P176A^ and *mrpL3*^P179A^ mutants respectively, indicated that their ability to perform respiratory-based growth at both 30 °C and 37 °C was similar to that of the WT MrpL3/mL44 control analyzed in parallel (Fig. S3). Likewise, mutation of residues towards the end of α-helix #1, that is, Lys(K)169 (to a negatively charged residue, *mrpL3*^K169D^) or the hydrophilic Ser(S)170 and the highly conserved aromatic residue Tyr(Y)175 to alanine residues (*mrpL3*^S170A^ and *mrpL3*^Y175A^, respectively) also did not adversely impact MrpL3/mL44’s ability to support respiratory growth at either temperature analyzed (Figs. S3 and 4*A*). Introduction of a positively charged residue at this position of MrpL3/mL44 through the mutation of Ile(I)171 to lysine (*mrpL3*^I171K^) also did not appear to prevent MrpL3/mL44 from supporting the aerobic respiratory of the cell, as growth of the *mrpL3*^I171K^ mutant on glycerol at both 30 °C and 37 °C mirrored that of the WT MrpL3/mL44 control (Fig. S3). Taking these results together, we conclude that residues K169, S170, I171, Y175, P176, P179, and A186, which represent conserved aspects of the α-helix#1-turn-α-helix#2 region of MrpL3/mL44, appear to be tolerant to individual mutation. As these *mrpL3* mutants did not exhibit any detectable respiratory growth phenotypes, we elected not to perform any further analysis of them but rather to focus on the next *mrpL3* mutants that did exhibit growth phenotypes.

**Figure 4:**
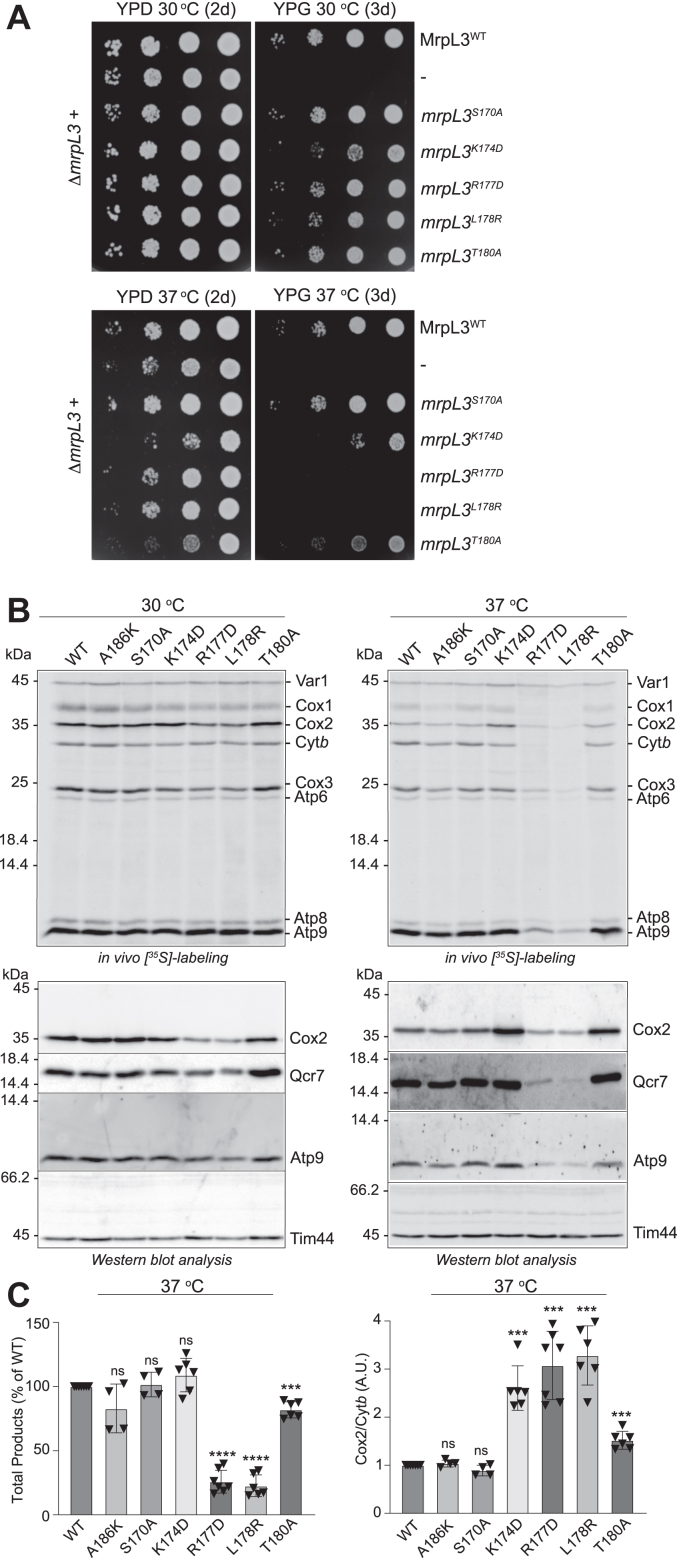
**Identification of residues R177 and L178 as critical for the function of yeast MrpL3/mL44***A*, growth phenotype analysis on glucose (YPD) (2d) or glycerol (YPG) (3d) of indicated strains was performed as described in Fig. 2*A*. *B*, *in vivo* radiolabeling of mitochondrial translation with [^35^S]methionine was performed in *mrpL3* mutant strains that had been adapted to galactose selective media and grown at 30 °C or 37 °C, as indicated. Following the radiolabeling pulse, total cellular proteins were extracted from each yeast strain and analyzed by SDS-PAGE analysis, Western blotting, autoradiography (*upper* panels), as described in Fig. 2*B*. Immunedecorations of the same cell extracts with antibodies directed against the indicated OXPHOS subunits (and Tim44, loading control) were performed as described in Fig. 2*B*. Note the exposure times for the OXPHOS subunit decorations in the 37 °C samples were 2 to 3 fold longer than that for the 30 °C samples, as the levels of OXPHOS complexes (especially of the COX complex) are lower in yeast cells grown at the higher temperature of 37 °C. The immunedecoration data of OXPHOS subunit levels at 37 °C was quantified from multiple independent Western blots and summarized in Fig. S4*A*. *C*, the sum of total radiolabeled products synthesized at 37 °C in each strain analyzed in *B* was quantified by phosphorimager analysis of resulting Western blots and, as described in Fig. 2*C*, expressed as a percentage of the WT control sum at that temperature (*left* panel). The ratio of newly synthesized Cox2/Cyt*b* in each of the mutants analyzed was calculated and expressed as a ratio of that measured in the WT control (A.U. arbitrary units) (*right* panel). Data from independent experiments and Western blots (n = 4–7) are provided. (A186K and S170A = four technical replicates from two independent experiments; K174D, L178R, and T180A mutants = six technical replicates from three independent experiments; R177D = seven technical replicates from four independent experiments.) Statistical analyses of total radiolabeled products synthesized and of the Cox2/Cyt*b* ratios, as compared to their respective WT control, were performed using an unpaired Welch’s *t* test; ns, not significant; ∗∗∗*p* = < 0.001, ∗∗∗∗*p* = < 0.0001. Bars indicate the means ± SD. WT, wild-type MrpL3/mL44; A186K, *mrpL3*^A186K^; S170A, *mrpL3*^S170A^; K174D, *mrpL3*^K174D^; R177D, *mrpL3*^R177D^; L178R, *mrpL3*^L178R^; T180A, *mrpL3*^T180A^.

Further mutagenesis of residues in MrpL3/mL44’s α-helix#1-turn-α-helix#2 region uncovered conserved aspects of the protein that were critical for the functionality of the MrpL3/mL44 protein. Mutation of charged Arg(R)177 of MrpL3/mL44 (equivalent to Asp(D)147 of human MRPL44/mL44) to a negatively charged residue proved to be strongly detrimental, as indicated by an inability of the resulting *mrpL3*^R177D^ mutant to grow on glycerol media at 37 °C (Fig. 4*A*). While exchange of hydrophobic residue Leu(L)178 of MrpL3/mL44 (and equivalent to residue Met(M)148 of the hydrophobic pocket in human MRPL44/mL44) to Ala(A), another hydrophobic residue (*mrpL3*^L178A^) did not appear to be problematic for function (Fig. S3), mutation of this residue to a positively charged residue arginine (R) was found to be detrimental for function (Fig. 4*A*). The resulting mutant *mrpL3*^L178R^ displayed a strong temperature-sensitive respiratory growth phenotype, that is, absence of growth on glycerol media at 37 °C (Fig. 4*A*). Respiratory-based growth of both the *mrpL3*^L178R^ and *mrpL3*^R177D^ mutants at 30 °C resembled that of the WT control, indicating that mutation of L178 and R177 did not completely inactivate MrpL3/mL44 protein but compromised its integrity at elevated temperatures (Fig. 4*A*). Finally, individual mutation of conserved residues K174 (to aspartic acid, *mrpL3*^K174D^) and Thr(T)180 (to alanine, *mrpL3*^T180A^) resulted in a partial respiratory growth phenotype at the elevated temperature of 37 °C (Fig. 4*A*). The *mrpL3*^K174D^ mutant exhibited reduced capacity for respiratory growth at 30 °C also. Interestingly, in contrast to the other *mrpL3* mutants analyzed in this study, the *mrpL3*^K174D^ and *mrpL3*^T180A^ mutants also displayed a growth phenotype on the fermentable carbon source glucose at 37 °C, suggesting the presence of these mutant *mrpL3* derivatives may exert a toxic effect on overall cell viability at the elevated stress temperatures.

To further characterize the *mrpL3*^K174D^, *mrpL3*^R177D^, *mrpL3*^L178R^, and *mrpL3*^T180A^ mutants, the strains were grown in galactose-containing media at both 30 °C and 37 °C. In contrast to glucose, which represses mitochondrial OXPHOS biogenesis, galactose is nonrepressing and while promoting OXPHOS complex assembly and respiration, it is not dependent on it to support yeast growth. The control WT MrpL3/mL44 and two of the nonphenotypic *mrpL3* mutants, *mrpL3*^A186K^ and *mrpL3*^S170A^, were also analyzed in parallel. *In vivo* radiolabeling in the presence of [^35^S]methionine (and cycloheximide) was used to measure the capacity of the mitoribosomes in each strain to perform new protein synthesis (Fig. 4, *B* and *C*). When grown at 30 °C, all mutants displayed a robust capacity for mitochondrial translation, resembling the WT MrpL3/mL44 control in translational levels, indicating that mutation of these residues did not render the MrpL3/mL44 protein totally ineffective. Mitochondrial translation activity was however strongly decreased in the most phenotypic *mrpL3*^R177D^ and *mrpL3*^L178R^ mutants when analyzed at 37 °C, with output levels in both mutants being reduced to 20 to 30% of the WT control. Mitochondrial translation was somewhat reduced in the *mrpL3*^T180A^ mutant when grown at 37 °C, with translational output levels approximately 80% of the WT control, whereas the overall mitotranslational levels of the *mrpL3*^K174D^ mutant appeared unaffected. The *mrpL3*^A186K^ mutant analyzed in parallel displayed a slight reduction in mitochondrial protein synthesis and corresponding partial reduction in OXPHOS levels when cultivated at 37 °C on galactose, in contrast to what was observed when grown under more stringent respiratory-based (glycerol) growth conditions (Fig. 2*B*). An alteration in the profile of the proteins synthesized in the *mrpL3*^K174D^, *mrpL3*^R177D^, *mrpL3*^L178R^, and *mrpL3*^T180A^ mutants was observed at 37 °C, which was most evident when the ratio of radiolabeled Cox2 to cytochrome *b* (Cyt*b*) synthesis was compared (Fig. 4*C*). The Cox2/Cyt*b* ratio was elevated not only in the *mrpL3*^K174D^ and *mrpL3*^T180A^ mutants relative to the WT control, but also in the *mrpL3*^R177D^ and *mrpL3*^L178R^ mutants, despite their overall reduced levels of protein synthesis (Fig. 4*C*). We conclude therefore that mutation of the hydrophobic pocket in MrpL3/mL44 in this manner resulted in mitoribosomes with altered transcript preference and/or translational efficiencies.

Consistent with their reduced capacity for both aerobic growth and mitochondrial translation, Western blot analysis of the cellular extracts indicated that steady state cellular OXPHOS levels were reduced in the *mrpL3*^R177D^ and *mrpL3*^L178R^ mutants grown at 37 °C, as indicated by the analysis of Cox2, Qcr7, and Atp9 protein levels relative to the WT and nonphenotypic mutants *mrpL3*^A186K^ and *mrpL3*^S170A^ analyzed in parallel (Fig. 4*B*, lower panels and Fig. S4*A*). BN-PAGE analysis of mitochondria isolated from the *mrpL3*^R177D^ and *mrpL3*^L178R^ mutants grown at 37 °C confirmed the strongly reduced levels of the cytochrome *bc*_1_ complex, COX, and F_1_F_o_-ATP synthase complexes in these mutants (Fig. S4*B*). Consistent with their retained capacity for some (albeit reduced) aerobic growth at 37 °C, the steady state levels of the cytochrome *bc*_1_ complex (Qcr7) and F_1_F_o_-ATP synthase (Atp9) in the *mrpL3*^K174D^ and *mrpL3*^T180A^ mutants were not as adversely impacted as they were in the more phenotypic *mrpL3*^R177D^ and *mrpL3*^L178R^ mutants. Interestingly, the steady state levels of Cox2 and the COX complex were significantly increased in the *mrpL3*^K174D^ and *mrpL3*^T180A^ mutants grown at 37 °C (Fig. 4*B*, lower panels and Fig. S4). BN-PAGE analysis indicated that the levels of assembled COX complex were noticeably elevated in the *mrpL3*^K174D^ mutant-isolated mitochondria in contrast to the levels of assembled F_1_F_o_-ATP synthase and cytochrome *bc*_1_ complex, which were similar or somewhat reduced, respectively, relative to the WT MrpL3/mL44 control (Fig. S4*B*). Together, these findings indicate that an imbalance in the stoichiometry of the COX complex relative to other OXPHOS complexes exists in the *mrpL3*^K174D^ and *mrpL3*^T180A^ mutants which displayed elevated levels of Cox2 synthesis.

Residues R177 and L178 of MrpL3/mL44 represent highly conserved residues among mL44 proteins. The hydrophobicity of the residue in a position equivalent to L178 is conserved among different mL44 proteins analyzed (*e.g.* Met(M)148 in human MRPL44/mL44). In the mL44 sequences analyzed, a charged residue is frequently located in a position equivalent to R177. In fungal mL44 proteins, this is often a positively charged residue, whereas in mammalian mL44 proteins, a negatively charged residue can often be found, for example, Asp(D)147 in the human MRPL44/mL44 protein (Fig. 1*A*). Analysis of the human MRPL44/mL44 cryoEM structure has indicated that the residue D147 is located in close proximity (3.7 Å) to the positively charged R248 residue (Fig. S5), suggesting they may represent a functionally interacting charged pair of residues in the human mL44 protein. In yeast MrpL3/mL44, the residue equivalent to human R248 is Glu(E)314, thus preserving the opposite charge nature of these potentially paired residues. Although the available cryoEM structure of MrpL3/mL44 would suggest that the corresponding residues (R177 and E314) in yeast are physically further apart than the equivalent residues in the human MRPL44/mL44 protein (Fig. S5), we cannot rule out that these residues may support each other if the MrpL3/mL44 protein were to exhibit dynamic conformation alterations, for example, during protein synthesis.

**Figure 5:**
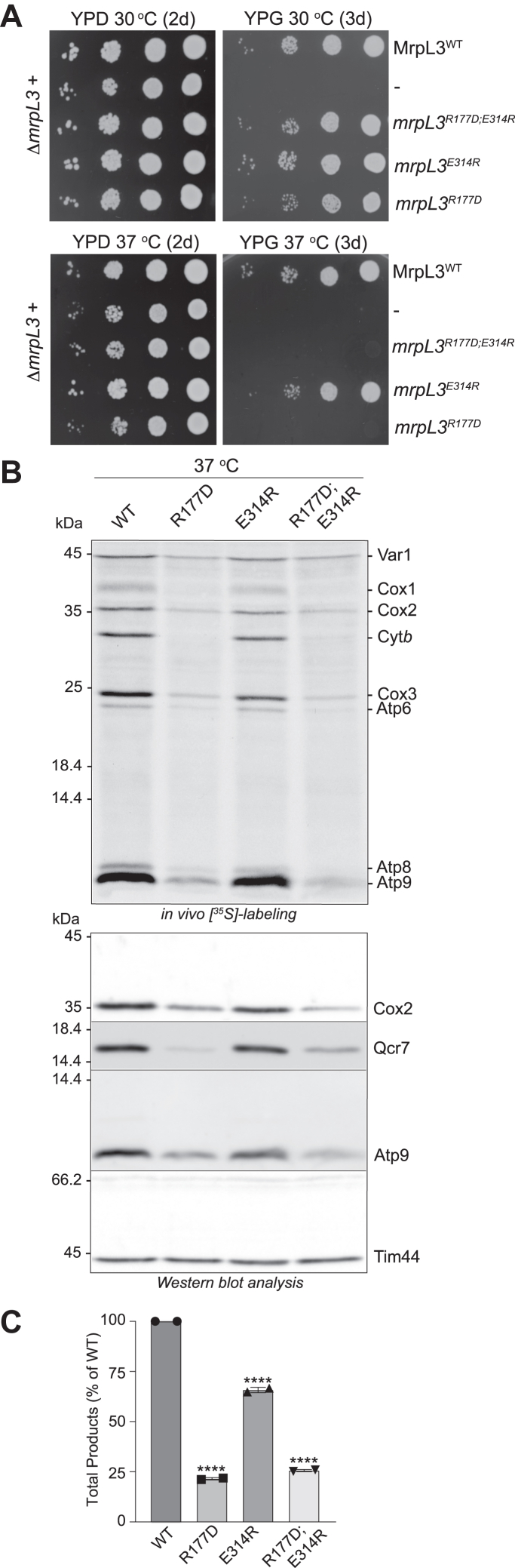
**Residue R177 of MrpL3/mL44 does not appear to functionally interact with conserved E314 residue.***A*, growth phenotype analysis on glucose (YPD) (2d) or glycerol (YPG) (3d) of indicated strains was performed as described in Fig. 2*A*. *B*, indicated *mrpL3* strains were grown on selective galactose media at 37 °C and *in vivo* labeling of mitochondrial translation with [^35^S]methionine, followed by extraction of total cellular proteins, SDS-PAGE analysis, Western blotting, autoradiography was performed, as described in Fig. 2*B*, *upper* panel. Steady state levels of OXPHOS proteins (Cox2, Qcr7, and Atp9) and Tim 44 (loading control) in these cell extracts were also analyzed following immunedecorations (*lower* panels). *C*, the sum of the total radiolabeled products synthesized in each strain analyzed in *B* was quantified by phosphorimaging analysis, as described in Fig. 2*C*. Data from technical replicates (n = 2) from two independent Western blots are provided. Statistical analysis of total radiolabeled products synthesized in each mutant compared to that of the WT control was performed using a one-way ANOVA with Dunnett’s *post hoc* test using GraphPad Prism 9, ∗∗∗∗*p* = < 0.0001. Bars indicate the means ± SD. WT, wild-type MrpL3/mL44; R177D, *mrpL3*^R177D^; E314R, *mrpL3*^E314R^; R177D;E314R, *mrpL3*^R177D;E314R^.

To test the possibility of a potential supportive interaction between residues R177 and E314 of yeast MrpL3/mL44, we mutated E314 to a positively charged residue (E314R) both as an individual mutation (*mrpL3*^E314R^) and in context of the R177D mutation (*mrpL3*^E314R;R177D^). If these residues functionally supported each other, we hypothesized that the E314R mutation alone would be detrimental to MrpL3/mL44’s function, that is, in a similar manner that the R177D mutation was, and that a double reverse charge mutation, that is, mutation of both R177 and E314 residues to reverse their charges in the same protein should (at least partially) restore function to the *mrpL3*^R177D^ mutant. Growth analysis however of the resulting mutants indicated that residue E314 was not critical to MrpL3/mL44’s function, as no obvious respiratory growth defect was observed at either 30 °C or 37 °C (Fig. 5*A*). Furthermore, the growth behavior of the double mutant, *mrpL3*^E314R;R177D^ closely resembled that of the single *mrpL3*^R177D^ mutant at 37 °C. *In vivo* radiolabeling analysis of mitochondrial translation in these mutants indicated that the strong translational defect observed in the *mrpL3*^R177D^ mutant at the elevated temperature of 37 °C persisted in the *mrpL3*^E314R;R177D^ mutant, indicating that double charge reverse mutation of E314 and R177 did not improve MrpL3/mL44’s ability to support mitoribosomal activity (Fig. 5, *B* and *C*). Mitochondrial translation was partially reduced in the *mrpL3*^E314R^ mutant at 37 °C but clearly not to the same extent as that measured in the *mrpL3*^R177D^ and *mrpL3*^E314R;R177D^ mutants. Consistently, cellular levels of OXPHOS complex marker subunits (Cox2, Qcr7, and Atp9) were strongly reduced in the *mrpL3*^R177D^ and *mrpL3*^E314R;R177D^ mutants and much less so in the *mrpL3*^E314R^ single mutants, relative to the WT control (Fig. 5*B*). On the bases of these results, we conclude that a negative charge in residue position 314 (E314) is not as important as the arginine in position 177 (R177), thereby suggesting that these residues in the yeast MrpL3/mL44 protein may not be physically interacting and supporting each other.

### Mutation of residues R177 and L178 of MrpL3/mL44 impairs assembly of the large ribosomal subunit

**Figure 6:**
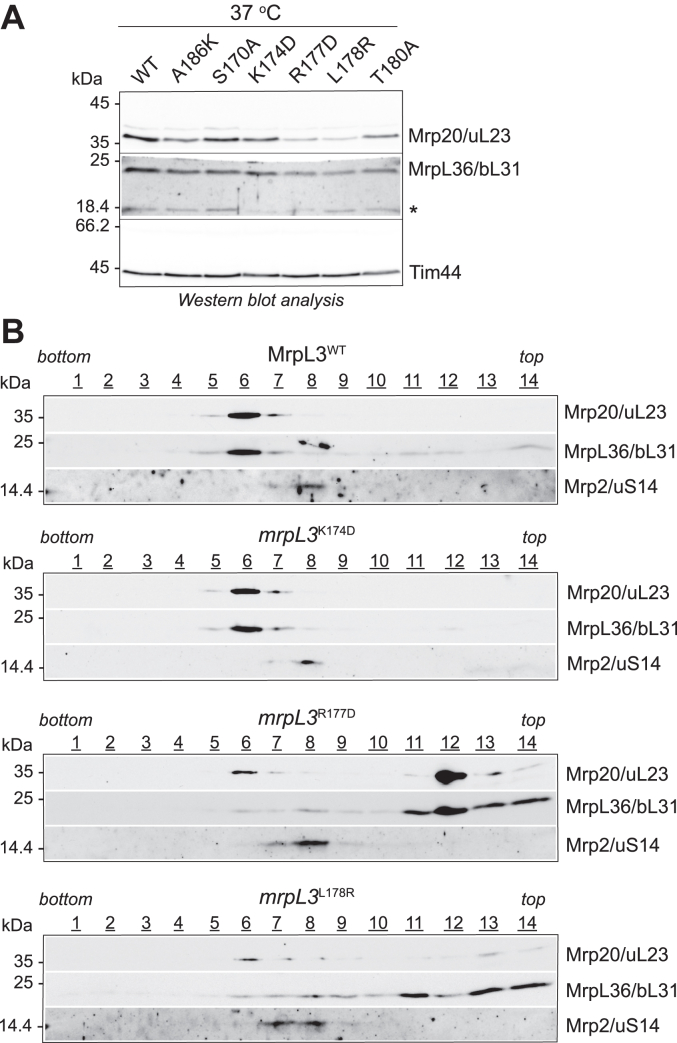
**The assembly of the large ribosomal subunit is perturbed in the *mrpL3***^**R177D**^**and *mrpL3***^**L178R**^**mutants.***A*, the indicated strains were grown on selective galactose media at 37 °C, following which total cell proteins were extracted and analyzed by SDS-PAGE and Western blotting. Immunedecoration of the resulting blot was performed with indicated mitoribosomal antibodies (Tim44 was analyzed as a loading control). The equivalent of A_600_ 0.3 of each cell type was loaded on the gel. Note the asterisk (∗) indicates a nonspecific signal observed following decoration with the horseradish peroxidase–coupled secondary antibody. Abbreviations, as in Fig. 4*B*. *B*, mitochondria were isolated from the *mrpL3*^K174D^, *mrpL3*^R177D^*, mrpL3*^L178R^ mutants, and the WT MrpL3/mL44 (MrpL3^WT^) strains, which had been grown in selective galactose media at 37 °C. The isolated mitochondria were first solubilized with octyl glucoside lysis buffer, and resulting extracts were fractionated by linear sucrose gradient analysis, as described in Experimental Procedures. Fractions (labeled from *bottom* to *top* of gradient) were TCA precipitated and analyzed by SDS-PAGE and Western blotting, followed by immunedecoration, as indicated. Antibodies against Mrp20/uL23 and MrpL36/bL31 were used to monitor the assembly state of the large mitoribosomal subunit. The assembly state of the small mitoribosomal subunit assembly state was monitored by immunedecoration with Mrp2/uS14 antibodies.

As the most phenotypic mutants, *mrpL3*^R177D^ and *mrpL3*^L178R^, displayed reduced capacity for mitotranslation, we explored if the levels and assembly state of mitoribosomes were compromised in these mutants, as MrpL3/mL44 has been previously shown to be critical for the early steps of mitoribosome assembly (31). Unfortunately, we failed in our efforts to generate an antibody against yeast MrpL3/mL44 (and the available human MRPL44/mL44 does not cross react with the yeast protein), so we could not directly assess if the levels of the *mrpL3*^R177D^ and *mrpL3*^L178R^ mutant proteins were limiting at elevated temperatures for mitoribosome assembly. Instead, levels of the mitoribosomal marker protein Mrp20/uL23 were analyzed and found to be reduced in the isolated *mrpL3*^R177D^ and *mrpL3*^L178R^ mitochondria grown at 37 °C, when compared to the *mrpL3*^K174D^ and WT MrpL3/mL44 control (Fig. 6*A*). The stability of Mrp20/uL23 is dependent on its ability to assemble into the large ribosomal (54S) subunit and thus serves as a sensitive marker for the 54S assembly levels in the cell (30, 36, 38). These findings are consistent with the possibility that the *mrpL3*^R177D^ and *mrpL3*^L178R^ mutant proteins may be compromised in their levels and/or ability to support mitoribosomal assembly when grown at the nonpermissive temperature.

To directly analyze the assembly state of the mitoribosomes, detergent extracts of the mitochondria isolated from the *mrpL3* mutant strains cultivated in galactose media at 37 °C were analyzed by sucrose gradient centrifugation (Fig. 6*B*). In WT MrpL3/mL44 mitochondrial extracts, the 54S ribosomal particle was largely recovered in fraction 6, as evidenced by the peak of both Mrp20/uL23 and MrpL36/bL31 signals. In contrast to Mrp20/uL23, the stability and hence the steady state levels of MrpL36/bL31 is not dependent on its mitoribosomal assembly, and thus MrpL36/bL31 represents a useful marker to analyze the behavior of mitoribosomal particles on sucrose gradients (30, 38, 39, 40). The assembly of the 54S particle in the *mrpL3*^K174D^ extract mirrored that of the WT control, as both Mrp20/uL23 and MrpL36/bL31 fractionated together with a peak in fraction 6. In contrast, the assembly of the 54S particle was perturbed in the *mrpL3*^R177D^ and *mrpL3*^L178R^ mutants, as evidenced by the recovery of the majority of MrpL36/bL31 in fractions towards the top of both gradients (where it was distributed largely between fractions 11–14). A small level of assembled 54S particles were observed in the *mrpL3*^R177D^ and *mrpL3*^L178R^ mitochondrial extracts, as evidenced by the cofractionation of a small amount of MrpL36/bL31 with the remaining Mrp20/uL23 protein in fraction 6 of these gradients. The presence of some assembled 54S particles in the *mrpL3*^R177D^ and *mrpL3*^L178R^ mitochondria (albeit strongly reduced in content) is consistent with the reduced, but detectable, level of translational capacity measured in these mutants at 37 °C (Fig. 4*B*). The small ribosomal (37S) subunit marker protein Mrp2/uS14 was found to consistently peak in fractions 7 to 8 of the *mrpL3* sucrose gradients, resembling the parallel WT MrpL3 control gradient. We conclude therefore that in contrast to the 54S particle, assembly of the 37S particle was not adversely impacted in the *mrpL3* mutants.

### MrpL3/mL44 residue Tyr(Y)165 located at the interface with MrpL15/mL57 is important for function

**Figure 7:**
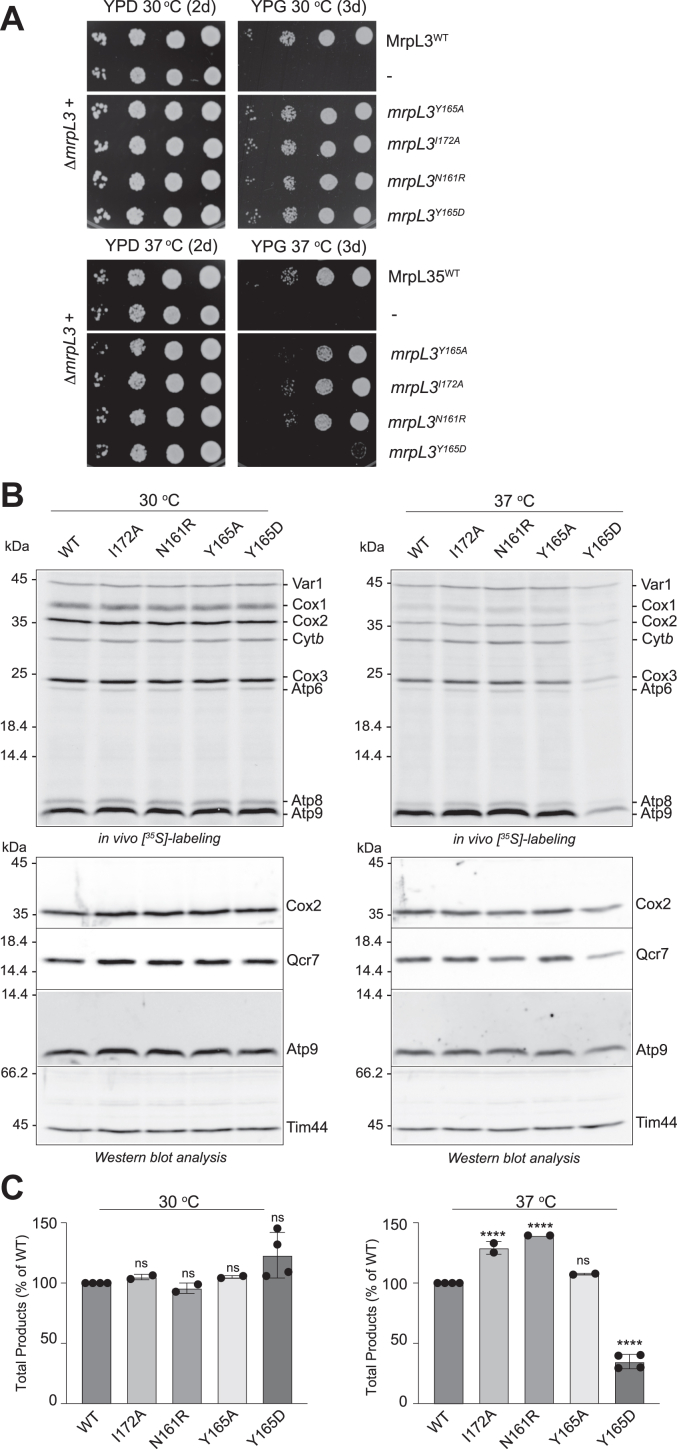
**Residue Y165 of yeast MrpL3/mL44 Y165, located at the interface with MrpL15/ml57, is important for the function**. *A*, growth phenotype analysis on glucose (YPD) (2d) or glycerol (YPG) (3d) of indicated strains was performed as described in Fig. 2*A*. *B*, mitochondrial translation capacity of indicated galactose-grown *mrpL3* mutants was determined through an *in vivo* radiolabeling approach with [^35^S]methionine, as described in Fig. 4*B*. Following the radiolabeling pulse, total cellular proteins were extracted from equivalent amounts of each strain and were subjected to SDS-PAGE analysis, Western blotting, and autoradiography (*upper* panels). Subsequent immunedecorations with indicated antibodies were performed as described in Fig. 4*B*. *C*, the sum of the total radiolabeled products synthesized in each strain analyzed in *B* was quantified by phosphorimager analysis, as described in Fig. 4*C*. With the exception of Y165D, data for each mutant is from technical replicates (n = 2) from two independent Western blots. Data from the Y165D mutant is from four technical replicates involving two independent experiments. Statistical analysis of total radiolabeled products synthesized in each mutant compared that of the WT control was performed using a one-way ANOVA with Dunnett’s *post hoc* test using GraphPad Prism 9; ns, not significant; ∗∗∗∗*p* = < 0.0001. Bars indicate the means ± SD. WT, wild-type MrpL3/mL44; I172A, *mrpL3*^I172A^; N161R, *mrpL3*^N161R^; Y165A, *mrpL3*^Y165A^; and Y165D, *mrpL3*^Y165D^.

Continuing with the mutagenesis of the α-helix#1-turn-α-helix#2 region of MrpL3/mL44, residues Asn(N)161, Tyr(Y)165, and Ile(I)172 (within the α-helix#1 region) were next selected for mutagenesis resulting in the creation of the *mrpL3*^N161R^, *mrpL3*^Y165A^, *mrpL3*^Y165D^, and *mrpL3*^I172A^ mutants. Each of these *mrpL3* derivatives were found to support aerobic growth of the Δ*mrpL3* strain at 30 °C and, with the exception of the *mrpL3*^Y165D^ mutant, also at 37 °C (Fig. 7*A*). The *mrpL3*^Y165D^ mutant in contrast to the others displayed an impaired capacity to grow at elevated temperatures on glycerol medium. Capacity for mitochondrial translation was reduced in the *mrpL3*^Y165D^ mutant at 37 °C, where it was determined to be approximately 30% of the MrpL3/mL44 WT control (Fig. 7, *B*, upper panel and *C*). Steady state levels of OXPHOS subunits (Cox2, Qcr7, and Atp9) were reduced accordingly in the *mrpL3*^Y165D^ (Fig. 7*B*, lower panel), indicating that the level of mitotranslation in this mutant was limiting to support efficient OXPHOS complex assembly at this temperature. We conclude from this analysis that the introduction of a charged residue into position Y165 was detrimental for the function of MrpL3/mL44 at elevated temperatures.

### Differential translation defects observed among transcripts in mrpL3 mutants

**Figure 8:**
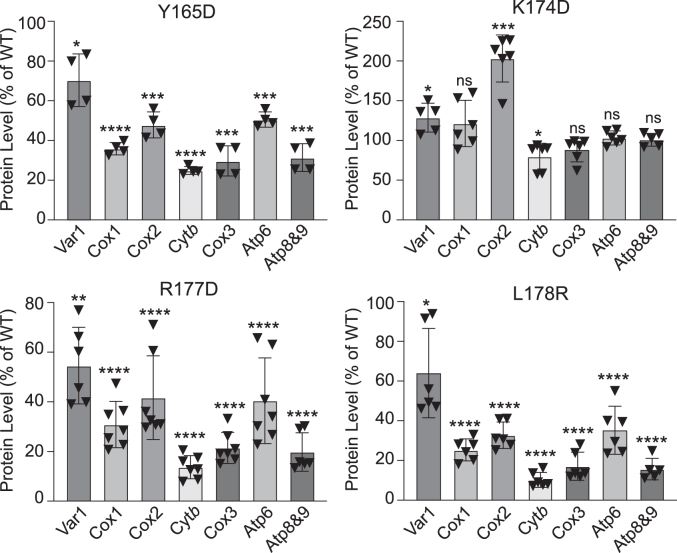
**The *mrpL3* mutant mitoribosomes display differential transcript preferences.** The synthesis levels of radiolabeled Var1, Cox1, Cox2, Cox3, Cyt*b*, Atp6, Atp8, and Atp9 proteins in the indicated *mrpL3* mutants were individually quantified. To do so, phosphorimaging analysis of the Western blots resulting from the *in vivo* radiolabeling experiments analyzed in Fig. 4, *B* and *C* (*mrpL3*^K174D^, *mrpL3*^R177D^, and *mrpL3*^L178R^ mutants) and Fig. 7, *B* and *C* (*mrpL3*^Y165D^ mutant) was performed to quantify the levels of each radiolabeled protein individually, with the exception of Atp8 and Atp9, which were quantified together, given the nature of their very close electrophoretic mobilities (Atp8&9). The level of each radiolabeled protein synthesized in indicated mutants was directly compared to (and expressed as a percentage of) its synthesis level in the WT MrpL3/mL44 control (WT) analyzed in parallel. For each protein, the statistical significance between its level synthesized in a specific *mrpL3* mutant and its parallel WT control was determined individually using a Welch’s *t* test. Data from independent experiments and Western blots (n = 4–7) are provided. (Y165D = four technical replicates from two independent experiments; K174D and L178R = six technical replicates from three independent experiments and R177D = seven technical replicates from four independent experiments.) Resulting significance/*p*-values are indicated: ns, not significant; ∗*p* = < 0.05; ∗∗*p* = < 0.01; ∗∗∗*p* = < 0.001; ∗∗∗∗*p* = < 0.0001. Bars indicate the means ± SD. Y165D*, mrpL3*^Y165D^; K174D*, mrpL3*^K174D^; R177D*, mrpL3*^R177D^; and L178R, *mrpL3*^L178R^.

Taken together, our mutagenesis approach has identified several residues (Y165, R177, and L178) within the α-helix#1-turn-α-helix#2 region of MrpL3/mL44 that appear critical for the protein’s ability to support mitoribosomes and their translational activity. Although significantly impaired, the *mrpL3*^Y165D^, *mrpL3*^R177D^, and *mrpL3*^L178R^ mutants retained some capacity for mitochondrial protein synthesis at 37 °C, consistent with the conclusion they still had a small population of assembled and functional ribosomes (Figs. 4*B* and 7*B*). The inhibitory effect of these *mrpL3* mutations on mitotranslation was observed not to be consistent across all of the mtDNA-encoded transcripts, however. Individual analysis of the levels of each of the radiolabeled proteins synthesized revealed that there was a differential inhibitory effect depending on the transcript being translated (Fig. 8). The synthesis of radiolabeled Var1 was consistently the least impaired of all transcripts, whereas the synthesis levels of Cyt*b*, Cox3, Atp8, and Atp9 were the most impacted of transcripts in these mutants. An intermediate effect was observed for the Cox1, Cox2, and Atp6 transcripts. Although the *mrpL3*^K174D^ mutant did not display a gross perturbation in overall translational output (Fig. 4*B*), a significant increase in Cox2 translation relative to other transcripts was recorded in this mutant. (Fig. 8). Together, our findings indicate that a mitoribosomal selectivity or prioritization for the translation of specific transcripts over others may exist in these *mrpL3* mutants.

## Discussion

In humans, the L156R mutation located in a conserved hydrophobic pocket region of the mitoribosomal MRPL44/mL44 protein resulted in clinical manifestation of childhood cardiomyopathy and progressive multisystem pathologies (13, 14, 15, 32, 33). In this study, we have investigated the feasibility of using *S. cerevisiae* as a model organism to study the molecular impacts of the disease-related mL44 protein. Our findings here indicate that the yeast and mammalian mL44 proteins have sufficiently diverged in evolution that the corresponding mutation in the yeast MrpL3/mL44 protein (the A186R or K mutation) did not confer any major adverse consequences for mitoribosomal synthesis capacity or MrpL3/mL44’s ability to support of OXPHOS-based cellular growth on glycerol. A small reduction in mitotranslation, and a corresponding minor decrease in OXPHOS complex levels, was however observed in the galactose-grown *mrpL3*^A186K^ mutant, suggesting the mitochondrial translation system was more impacted by this mutation under fermentation growth conditions. The disease-related residue L156 in mammalian MRPL44/mL44 is located in a conserved hydrophobic pocket of the protein formed by two interfacing helix regions, termed here the α-helix#1-turn-α-helix#2 region. Results presented here indicate that aspects of this hydrophobic pocket region are critical for MrpL3/mL44’s ability to support mitoribosome assembly and to ensure normal translational output. Residues Y165, R177, and L178 in this pocket were found to be critical for MrpL3/mL44’s functional integrity. Mutation of these residues to charged ones (or to an opposite charge in the case of R177) resulted in temperature-sensitive phenotypes that compromised MrpL3/mL44’s ability to support mitotranslation and consequently OXPHOS assembly and respiratory growth at elevated temperatures. Yeast mitoribosomal assembly studies have indicated that MrpL3/mL44 together with other partner membrane protuberance proteins (MrpL13/m50, MrpL15/m57, and MrpL20/ml58) form an early nucleation site for the hierarchical process of mitoribosomal assembly (31). The presence of MrpL3/mL44 is thus critical for ensuring the effective assembly of productive mitoribosomes and their tethering to the membrane surface. Although we cannot directly assess the levels of the *mrpL3* mutant derivative proteins in these mutants (due to the absence of a functioning MrpL3/mL44 antibody), we consider it possible that the Y165, R177, and L178 mutations may render the *mrpL3* proteins structurally unstable and proteolytically vulnerable at elevated temperatures causing them to be limiting for function. Consequently, limiting MrpL3/mL44 levels, functional and/or physical, are proposed here to primarily underlie the observed defects in mitoribosomal assembly levels and consequently translational output in these conserved hydrophobic pocket *mrpL3* mutants. Furthermore, our data would support that there may be a functional diversity or hierarchy to mitochondrial ribosomes, such that the translational transcript preference or efficiency of translation may not be equal across the entire population of mitoribosomes. Our findings here indicated that the Y165, R177, and L178 *mrpL3* mutations did not equally impair the translation of all mitochondrial transcripts. The translationally active mitoribosomes remaining in the *mrpL3*^Y165D^, *mrpL3*^R177D^, and *mrpL3*^L178R^ mutants displayed a leading preference for translating the mitoribosomal Var1 protein. These mutants also appeared to favor the translation of Cox1, Cox2, and Atp6 transcripts over those of Cox3, Cyt*b*, Atp8, and Atp9. Prioritizing the Var1 transcript presumably serves to ensure the supply of functioning mitoribosomes to the cell, as Var1 is an essential component of the small mitoribosomal subunit.

In addition to identifying residues in MrpL3/mL44 critical to maintaining functional levels of the protein, our analysis has uncovered two other residues in MrpL3/mL44, K174 and T180, which have the capacity to modulate the translational profile of the mitoribosomes. While mutation of K174 and T180 did not grossly impact the overall translational output of the mitoribosomes, we report here that an elevated level of Cox2 new protein synthesis was observed in the *mrpL3*^K174D^ and the *mrpL3*^T180A^ mutants. The imbalance of Cox2 transcript translation in these mutants also manifested into elevated steady state levels of the assembled COX complex (relative to WT control) such that a stoichiometric imbalance of OXPHOS complexes was observed, as revealed through BN-PAGE analysis of the *mrpL3*^K174D^ mutant. To our knowledge, the existence of a mitoribosomal mutant where the cellular COX levels exceed those of the WT control is a novel finding in the literature. Furthermore, our findings would suggest that a stoichiometric excess of COX complex relative to other OXPHOS enzymes could be partially toxic to the cell, as impaired growth also under fermentation (glucose) conditions was observed in both of the *mrpL3*^K174D^ *mrpL3*^T180A^ mutants. Elevated Cox2 synthesis (*e.g.* relative to Cyt *b*) was also observed in the *mrpL3*^R177D^ and *mrpL3*^L178R^ mutants, despite their overall reduction in mitotranslational capacity. Together, it would appear that mutation of the hydrophobic pocket region of MrpL3/mL44 manifested in a misregulation of Cox2 synthesis, which may normally need to be kept in strict check with other transcripts due to a detrimental impact of elevated Cox2 synthesis and COX levels on cell viability.

We demonstrate here that the human MRPL44/mL44 protein cannot functionally replace the yeast MrpL3/mL44 protein, highlighting that the structure and function of the mL44 proteins must have diverged over evolution. While cryoEM analysis of the human and yeast mitoribosomes has revealed similarities in their membrane protuberance regions, the fungal mitoribosome has been shown to contain two additional proteins MrpL15/mL57 and MrpL20/mL58, not found in their mammalian counterparts (17, 18, 20, 26, 27). MrpL15/mL57 and MrpL3/mL44 are both vestigial members of double-stranded RNA-binding exonuclease III protein family, which form a close association with each other and with a fungal-specific expansion of the 21S rRNA, termed helix 0-ES1/2 (17) (Fig. S1*A*). MrpL3/mL44 interfaces with MrpL15/mL57 in a manner which involves more N-terminal aspects of the α-helix#1 region of MrpL3/mL44’s α-helix#1-turn-α-helix#2 region. The side chain of residue Y165 is exposed on the exterior α-helical surface of MrpL3/mL44 interfacing with MrpL15/mL57, whereas the side chains of the other nonphenotypic N161, I172 residues face internally within the MrpL3/mL44 protein. The *mrpL3*^Y165D^ mutation was deleterious for function, suggesting this mutation may interfere with MrpL3/mL44’s ability to functionally interact with MrpL15/mL57, highlighting the potential significance of this interaction for the function of the membrane protuberance region of the mitoribosome.

In summary, our findings here shed light on the importance of the conserved hydrophobic pocket–forming region of the mL44 proteins. Our data indicate the importance of this region for both the functional stability of the mL44 protein and its ability to support the stable assembly of the mitoribosomes, as well as the translational preference of the mitoribosome for certain mtDNA-encoded transcripts. Furthermore, although highly conserved, our data indicate an evolutionary divergence within the mL44 protein family and shed light on the functional significance of the yeast MrpL3/mL44’s interaction with the fungal-specific MrpL15/mL57 protein, an external facing component of the membrane protuberance region of the mitoribosome. Future analysis will be required to dissect the importance of this protein for the function and membrane association of the fungal mitoribosome.

## Experimental procedures

### Yeast strains and growth conditions

All *S. cerevisiae* strains in this study are in the haploid W303-1A genetic background (W303-1A, *mat a, leu2, trp1, ura3, his3, ade2*). Strains include the Δ*mrpL3* mutant (*MRPL3::KAN*) mutant and those harboring pRS413 plasmid borne copies of the WT MRPL3 gene or *mrpL3* mutant derivatives, constructed through a plasmid shuffling approach in the presence of YEplac112-*RNR1* plasmid (31) and as described below. Yeast strains were cultured using standard protocols on either synthetically defined (S) minimal medium supplemented as appropriate with uracil, tryptophan, leucine, adenine, histidine and appropriate carbon source (glucose (2%), SD; galactose (2%), SGal; or glycerol (2%)/ethanol (3%), SGE) or on full yeast extract/peptone media (supplemented with glucose (2%), YPD; glycerol (2%), YPG; or glycerol (2%)/ethanol (3%), YPEG), as indicated. Mitochondria were isolated from indicated strains grown at 37 °C in selective SGal medium.

### Generation of mrpL3 mutants

The WT MRPL3/mL44 ORF and 5’ (440 bp) and 3’ (158 bp) of the endogenous MRPL3 gene regulatory regions were amplified by PCR from yeast genomic DNA and cloned into both the pRS316 (URA3) and pRS413 (HIS3) centromeric plasmids resulting in pRS316-MRPL3 and pRS413-MRPL3, respectively. PCR-based site-directed mutagenesis was then used to create the indicated *mrpL3* mutants using the pRS413-MRPL3 plasmid as template. Correct mutation of targeted codon(s) was verified by sequencing the entire *mrpL3* gene insert.

To phenotypically analyze the created MrpL3/mL44 derivatives from the resulting pRS413-MRPL3/*mrpL3* plasmids, a yeast genetic system using a plasmid shuffling approach was employed. First, the yeast chromosomal MRPL3 ORF was entirely replaced with a PCR-amplified kanamycin resistance gene (KAN) cassette through homologous recombination into the MRPL3 locus in a haploid WT (W303-1A) yeast strain that harbored both the pRS316-MRPL3 and YEplac112-RNR1 (TRP1) plasmids. Overexpression of the RNR1 gene, which encodes a subunit of the dNTP checkpoint enzyme ribonucleotide reductase, has been previously described to suppress the instability of mtDNA that yeast strains deficient in mitoribosomal activity commonly experience (31). The resulting strain (Δ*mrpL3*+pRS316-MRPL3+ YEplac112-RNR1, URA+, TRP+, KAN+) was then transformed with the pRS413 plasmid harboring either the WT MRPL3 genomic insert (pRS413-MRPL3) or one bearing a mutant *mrpL3* gene derivative (pRS413-*mrpL3*) or no insert (*i.e.* the “empty” pRS413 plasmid). Following the plasmid shuffling process, involving growth on 5′-fluoroorotic acid (which promotes the loss of the pRS316-MRPL3 plasmid due to the toxicity of URA3-positive cells), Δ*mrpL3* transformants harboring only the pRS413 and YEplac112-RNR1 plasmids were selected. The ability of the mutated *mrpL3* derivatives to functionally replace the WT MrpL3/mL44 protein was then assessed by comparing the resulting Δ*mrpL3+*pRS413-MRPL3 (WT MrpL3/mL44) and Δ*mrpL3+*pRS413-*mrpL3* (mutant) strains, both phenotypically (growth assays) and biochemically. Note, this genetic system with the use of a centromeric plasmid (pRS413) and employment of MRPL3’s endogenous 5′ and 3′ regulatory regions to drive expression of MRPL3/*mrpL3* ORFs was designed to mimic the expression of the mutated protein derivatives at levels similar to the endogenous MrpL3/mL44 protein. A list of all the *mrpL3* mutants created and analyzed in this study is presented in Table S1.

### Expression of the human MRPL44/mL44 protein

The entire hMRPL44 ORF, translational stop codon, and engineered 5′ and 3′ restriction sites were initially synthesized as a 1028 bp gBlock gene fragment by Integrated DNA Technologies and used as template for PCR. The PCR-amplified hMRPL44 ORF was cloned into a multicopy yeast expression plasmid pVT100U-ADH1 (URA3) plasmid (37), engineered to contain the constitutive ADH1 5′ promoter and 3′-terminator regulatory regions, and sequence verified. The resulting plasmid (pVT100U-ADH1-MRPL44) was also introduced into W303-1A WT yeast cells, and the expression of MRPL44 was verified and determined not to be detrimental to the respiratory growth of the otherwise WT cell. The endogenous MRPL3 gene locus subsequently was disrupted in the WT+pVT100U-ADH1-MRPL44 strain through homologous recombination (KAN cassette and verified by analytical genomic PCR) and after the introduction of the YEplac112-RNR1 plasmid. Expression of hMRPL44 in the resulting strain, Δ*mrpL3*+pVT100U-ADH1-MRPL44+YEplac112-RNR1 strain, was confirmed through Western blotting of mitochondria isolated from the strain, prior to testing whether expression of hMRPL44 could complement the respiratory deficiency caused by the absence of MrpL3/mL44.

### Sucrose gradient analysis of mitoribosomes

Mitochondria were isolated from the indicated *mrpL3* mutant strains grown on SGal selective media at 37 °C. Sucrose gradient analysis was performed to determine the assembly states of the 54S and 37S mitoribosomal particles, essentially as previously described (38). Briefly, the isolated mitochondria (450 μg) were solubilized in 600 μl lysis buffer (50 mM Tris–HCl, 150 mM NaCl, 5 mM MgCl_2_, 1 mM PMSF, 1.5% octyl β-D-glucoside, pH 7.2) for 30 min on ice. Following a clarifying spin, the supernatant was layered onto a 10.5 ml linear sucrose gradient (25–40%) and centrifuged at 26,000 rpm for 18 h at 4 °C in Beckman SW-41 Ti rotor. Fractions were collected, TCA precipitated, and analyzed with SDS-PAGE and Western blotting with antibodies against 54S (Mrp20/uL23 and MrpL36/bL31) and 37S (Mrp2/uS14) marker mitoribosomal proteins.

### Reproducibility and statistics

Radiolabeling experiments, Western blot/immunodecorations, and serial dilution phenotype assays shown are typically representatives of multiple independent studies (see figure legends for more details). When indicated, statistical analysis was performed with GraphPad Prism 9 (GraphPad Software, https://www.graphpad.com/features) and the means and SDs are provided. Significance analysis (one-way ANOVA or unpaired Welch’s *t* test, as indicated) is also provided.

### Miscellaneous

Mitochondrial isolation, protein determination, SDS-PAGE, and Western blotting were performed as previously described (41). For the *in vivo* analysis of mitochondrial translation activity, mitochondrial gene products were labeled with [^35^S]methionine in the presence of cycloheximide (to inhibit cytosolic translation), as previously described (35, 36, 38, 42, 43). Following SDS-PAGE/Western blotting analysis, quantifications of radioactive signals were performed by STORM phosphorimaging of resulting Western blots and subsequent analysis using ImageQuant TL 10.2 analysis software (Cytiva Life Sciences, cytivalifesciences.com). The antibodies against Qcr7, Mrp20/uL23, (both gifts, see Acknowledgments), Cox3 (Invitrogen/Novex, Anti-Cox3 monoclonal #459300), and human MRPL44 (Proteintech, Rosemont, IL cat # 16394-1-AP) are all monoclonal antibodies. The Mrp2/uS14 antisera, a rabbit polyclonal, was a gift (see Acknowledgments). The antibodies against Cox2, cytochrome *c*_1_, and Tim44 were rabbit polyclonal and generated against the respective purified yeast proteins. The F_1_-α/β antibodies were chicken antibodies and also raised against the purified proteins. Specificity of these antisera had been previously controlled using tagged derivatives of the respective proteins. Antigen–antibody complexes on Western blots were detected by horseradish peroxidase–coupled secondary antibodies and chemiluminescence detection on X-ray films or using the Amersham Imager 600. When indicated, images were quantified using ImageJ software (imagej.net).

## Data availability

All data are contained within the manuscript.

## Supporting information

This article contains supporting information (Figs. S1–S5 and Table S1).

## Conflict of interest

The authors declare that they have no conflicts of interest with the contents of this article.
