# Supplementary material for: Importance of conserved hydrophobic pocket region in yeast mitoribosomal mL44 protein for mitotranslation and transcript preference
Source: J Biol Chem. 2024 Jun 29;300(8):107519. doi: 10.1016/j.jbc.2024.107519 (PMC11345376; doi:10.1016/j.jbc.2024.107519)
Supplement: Supporting information [file mmc1.pdf]

Supporting Information

Importance of conserved hydrophobic pocket region in yeast mitoribosomal mL44 protein for mitotranslation and transcript preference

Box, J.M., Higgins, M.E. & Stuart, R.A.

Materials included (7 pages total, S1-S7):

Figures S1-S5

Table S1

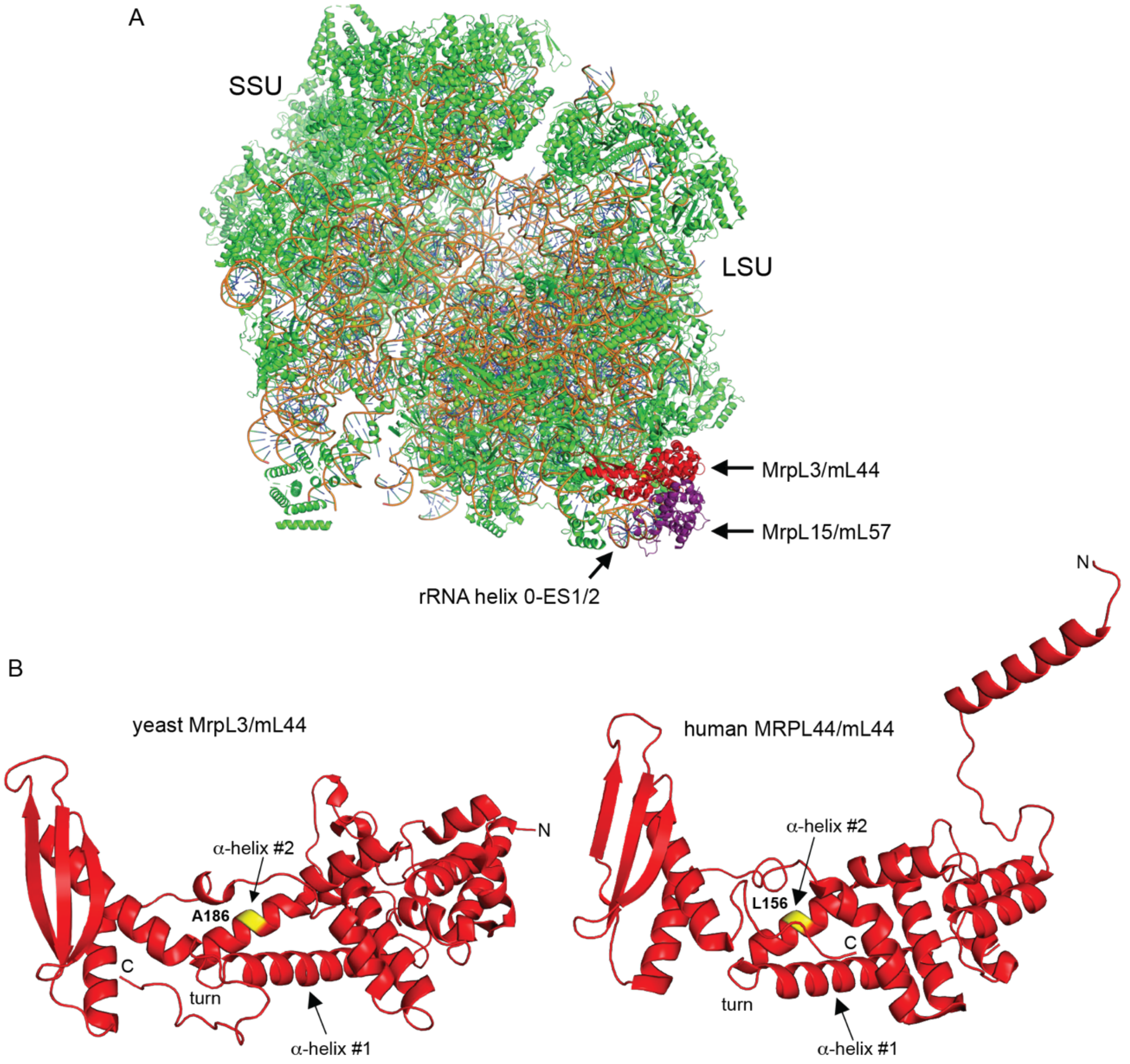

Figure S1: Structural features of yeast MrpL3/mL44 and human MRPL44/mL44 mitochondrial ribosomal proteins. *A*, *S. cerevisiae* mitochondrial ribosome small subunit (37S, SSU) and large subunit (54S, LSU) (from PDB 5MRC) with MrpL3/mL44 (red), MrpL15/mL57 (deep purple) and rRNA helix 0-ES1/2 highlighted on the membrane protuberance region of the LSU. *B*, Structural homology of yeast (*S. cerevisiae*) MrpL3/mL44 (PDB 5MRC, chain 5) compared to human (*H. sapiens*) MRPL44/mL44 (PDB 3J9M, chain c). The location of the disease-related residue (L156R) in the human MRPL44/mL44 protein, and the equivalent residue (A186) in yeast MrpL3/mL44, are highlighted in yellow on the α-helix #2 portion of the "α-helix#1-turn-α-helix#2" region for each protein. The N- and C- termini of both proteins are indicated by N and C, respectively.

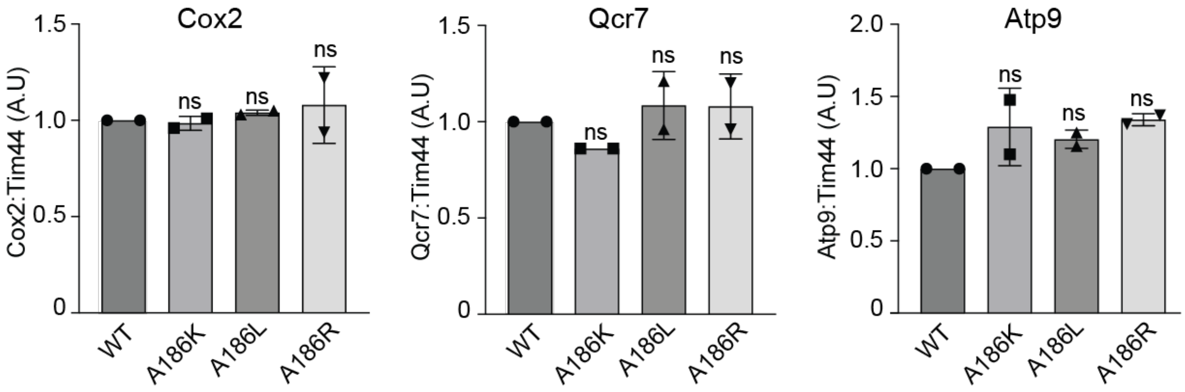

Figure S2: The *mrpL3* A186 mutants do not display a gross perturbation in OXPHOS steady state levels. The *mrpL3*^{A186K}, *mrpL3*^{A186L} and *mrpL3*^{A186R} strains were grown in glycerol containing media at 37 °C and used for *in vivo* radiolabeling to determine mitotranslation output, as described in Fig. 1B. The same cell extracts were analyzed by SDS-PAGE, Western blotting and immunedecoration with OXPHOS subunit specific antibodies (Cox2, Qcr7, Atp9) and Tim44 as a loading control. The chemiluminescent image signals from Cox2, Qcr7, Atp9 and Tim44 were quantified by ImageJ analysis, and the Cox2:Tim44, Qcr7:Tim44 and Atp9:Tim44 content ratios for each *mrpL3* strain were normalized to their corresponding wild-type (WT) control. A.U., arbitrary units. The OXPHOS subunit:Tim44 data from independent Western blots (n = 2) were quantified and summarized, as indicated. The significance between measurements of each OXPHOS subunit in the *mrpL3* mutant stain to its respective WT control was determined by one-way ANOVA with Dunnett's post hoc test using GraphPad Prism 9 and determined to be not significant (ns) in each case. Bars indicate the means +/- SD. Abbreviations: A186K, *mrpL3*^{A186K}; A186L, *mrpL3*^{A186L}; A186R, *mrpL3*^{A186R}.

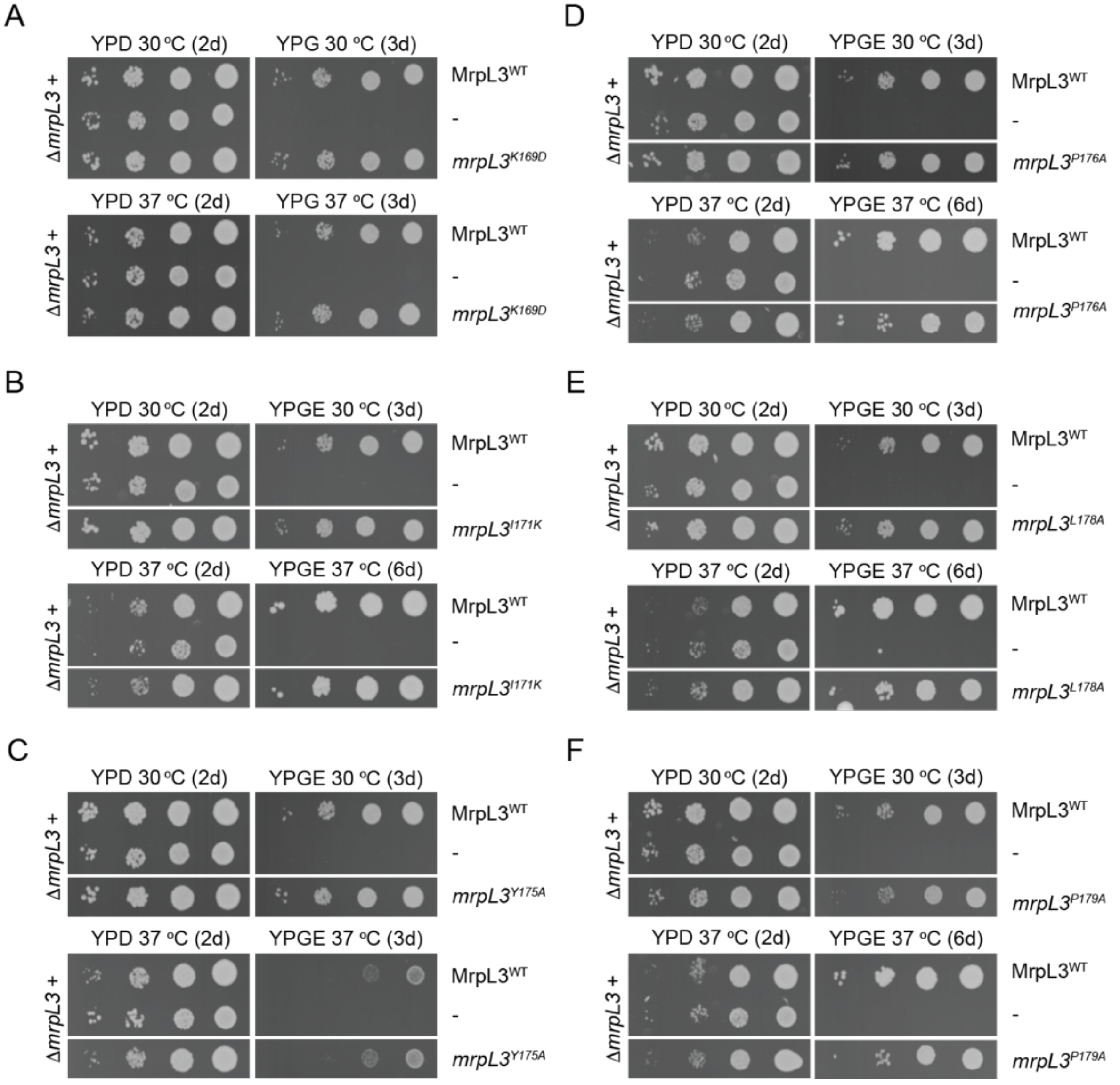

Figure S3: Mutation of some conserved residues of MrpL3/mL44 did not yield a discernable respiratory growth phenotype. *A-F*, 10-fold serial dilutions (right to left) of Δ*mrpL3* strains harboring the pRS413 plasmid containing no insert (-), or a gene insert encoding the wild-type MrpL3/mL44 protein (MrpL3^{WT}) or a mutated *mrpL3* derivative, as indicated. Strains were taken from 24 hr. growths on selective SD plates and spotted onto YP plates containing either glucose (YPD) (2d) or glycerol (YPG) (3d) or glycerol/ethanol (YPGE) (3d or 6d, as indicated) and incubated at either 30 °C or 37 °C.

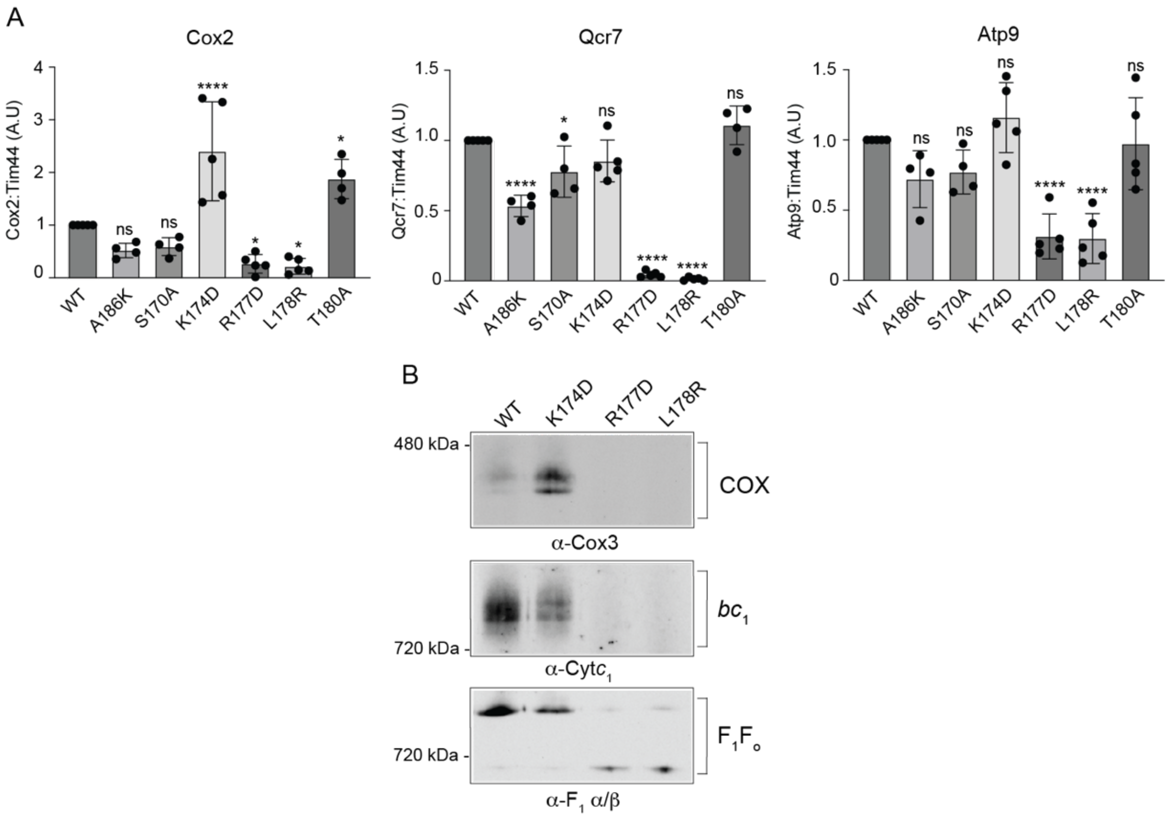

Figure S4: The *mrpL3*^{K174D} and *mrpL3*^{T180A} mutants contain increased Cox2 complex steady state levels. *A*, The total cell extracts prepared from the 37 °C-grown *mrpL3* strains used for *in vivo* radiolabeling in Fig. 3B and analyzed by SDS-PAGE and Western blotting, were immunedecorated with Cox2, Qcr7, and F$_o$- subunit Atp9 antibodies. Tim44 was also used as a loading control. The resulting chemiluminescent signals were quantified using Image J analysis. The steady state levels of Cox2, Qcr7 and Atp9 (all expressed as a ratio of Tim44) were quantified in each of the mutants and statistically compared to the WT control analyzed in parallel, using one-way ANOVA analysis with Dunnett's post hoc test using GraphPad Prism 9. Data from independent Western blots for each of these analyses (n=4-7) as described in Fig.4, are provided. The resulting statistical analysis was determined as: ns, not significant; * p = < 0.05; or **** p = ≤ 0.0001. Bars indicate the means +/- SD. *B*, Mitochondria were isolated from the indicated strains grown in galactose-media at 37 °C and were solubilized with 0.6 % dodecylmaltoside and subjected to BN-PAGE analysis. The resulting Western blots were immunodecorated with antibodies against Cox3 (α-Cox3), cytochrome c_1 (α-Cytc_1), and F$_1$-α/β (α-F$_1$-α/β), subunits of the COX, cytochrome bc_1 (bc_1) and F$_1$F$_o$-ATP synthase (F$_1$F$_o$) complexes, respectively. Other abbreviations: WT, wild-type MrpL3/mL44; A186K, *mrpL3*^{A186K}; S170A, *mrpL3*^{S170A}; K174D, *mrpL3*^{K174D}; R177D, *mrpL3*^{R177D}; L178R, *mrpL3*^{L178R}; T180A, *mrpL3*^{T180A}.

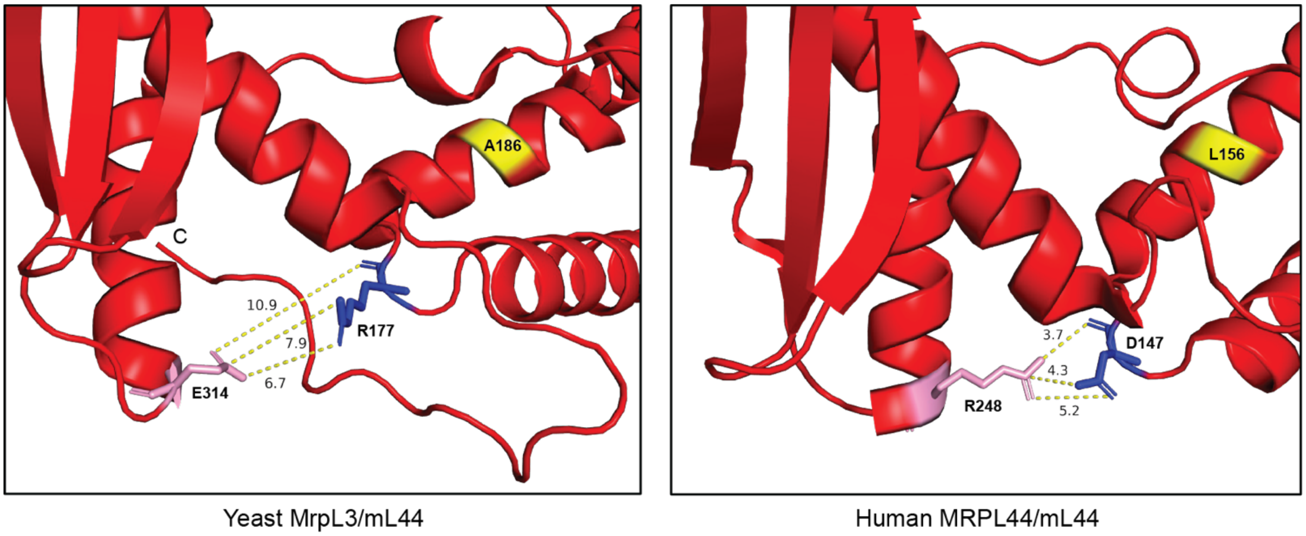

Figure S5: PyMOL images indicating the proximity of residues R177 and E314 of MrpL3/mL44 and D147 and R248 of human MRPL44/mL44. PyMOL analysis of yeast MrpL3/mL44 protein (from PDB 5MRC) and the proximity of residues (approximate Å distances indicated) R177 and E314 residues (left panel). Analysis of corresponding residues in the human MRPL44/mL44 protein (from PDB 3J9M), D147 and R248 (right panel). The positions of the disease-related residue L156 in human MRPL44/mL44 protein and its corresponding residue A186 in the yeast MrpL3/mL44 protein are indicated, yellow highlighted residue.

Location of residue within "α-helix #1-turn- α-helix #2"	*S.c. MrpL3/mL44 residue*	*Mutagenesis performed*	*Observed respiratory growth*
α-helix #1	Asn(N)161	N161R	normal @ 30 °C & 37 °C
α-helix #1	Tyr(Y)165	Y165A<br>Y165D	normal @ 30 °C & slightly reduced @ 37 °C<br>normal @ 30 °C & strongly reduced @ 37 °C
α-helix #1	Lys(K)169	K169D	normal @ 30 °C & 37 °C
α-helix #1	Ser(S)170	S170A	normal @ 30 °C & 37 °C
α-helix #1	Ile(I)171	I171K	normal @ 30 °C & 37 °C
α-helix #1	Ile(I)172	I172A	normal @ 30 °C & 37 °C
α-helix #1	Lys(K)174	K174D	somewhat reduced @ 30 °C & slightly reduced 37 °C
α-helix #1	Tyr(Y)175	Y175A	normal @ 30 °C & 37 °C
turn	Pro(P)176	P176A	normal @ 30 °C & 37 °C
turn	Arg(R)177	R177D<br>R177D; E314R	normal @ 30 °C & not detectable @ 37 °C<br>normal @ 30 °C & not detectable @ 37 °C
turn	Leu(L)178	L178A<br>L178R	normal @ 30 °C & 37 °C<br>normal @ 30 °C & not detectable @ 37 °C
turn	Pro(P)179A	P179A	normal @ 30 °C & 37 °C
turn	Thr(T)180	T180A	normal @ 30 °C & somewhat reduced 37 °C
α-helix #2	Ala(A)186	A186K<br>A186R<br>A186L	normal @ 30 °C & 37 °C<br>normal @ 30 °C & 37 °C<br>normal @ 30 °C & 37 °C
N-term prior to β-fold region	Glu(E)314	E314R<br>R177D; E314R	normal @ 30 °C & 37 °C<br>normal @ 30 °C & not detectable @ 37 °C

Table S1: Residues targeted in *S. cerevisiae* (*S.c.*) MrpL3/mL44 for mutagenesis and their location within the defined "α-helix #1-turn-α-helix #2" region. The mutagenesis performed column indicates which residues in MrpL3/mL44 were targeted and how they were mutated. In most cases residues were mutated to Ala(A), but in some instances they were mutated to a charged residue [Asp(D) or Arg(R)]. The mutated *mrpL3* protein derivatives were expressed in Δ*mrpL3* strain and the observed respiratory growth behavior of the resulting mutant strains at both 30 °C and 37 °C is indicated.
